# Immunoinformatic Identification of Multiple Epitopes of gp120 Protein of HIV-1 to Enhance the Immune Response against HIV-1 Infection

**DOI:** 10.3390/ijms25042432

**Published:** 2024-02-19

**Authors:** Arslan Habib, Yulai Liang, Xinyi Xu, Naishuo Zhu, Jun Xie

**Affiliations:** 1 Laboratory of Molecular Immunology, State Key Laboratory of Genetic Engineering, School of Life Sciences, Fudan University, Shanghai 200433, China; 20110700169@fudan.edu.cn (A.H.); 18110700002@fudan.edu.cn (X.X.); nzhu@fudan.edu.cn (N.Z.); 2 Institute of Biomedical Sciences, School of Life Sciences, Fudan University, Shanghai 200438, China

**Keywords:** HIV-1, gp120, in silico, epitope

## Abstract

Acquired Immunodeficiency Syndrome is caused by the Human Immunodeficiency Virus (HIV), and a significant number of fatalities occur annually. There is a dire need to develop an effective vaccine against HIV-1. Understanding the structural proteins of viruses helps in designing a vaccine based on immunogenic peptides. In the current experiment, we identified gp120 epitopes using bioinformatic epitope prediction tools, molecular docking, and MD simulations. The Gb-1 peptide was considered an adjuvant. Consecutive sequences of GTG, GSG, GGTGG, and GGGGS linkers were used to bind the B cell, Cytotoxic T Lymphocytes (CTL), and Helper T Lymphocytes (HTL) epitopes. The final vaccine construct consisted of 315 amino acids and is expected to be a recombinant protein of approximately 35.49 kDa. Based on docking experiments, molecular dynamics simulations, and tertiary structure validation, the analysis of the modeled protein indicates that it possesses a stable structure and can interact with Toll-like receptors. The analysis demonstrates that the proposed vaccine can provoke an immunological response by activating T and B cells, as well as stimulating the release of IgA and IgG antibodies. This vaccine shows potential for HIV-1 prophylaxis. The in-silico design suggests that multiple-epitope constructs can be used as potentially effective immunogens for HIV-1 vaccine development.

## 1. Introduction

HIV-1 and HIV-2 are two retroviruses that cause AIDS in humans [1]. These viruses have various similarities in terms of replication, transmission, and clinical signs [2]. HIV-1 is the primary cause of HIV infections globally, resulting in 680,000 fatalities in 2020 and a total of 37.7 million individuals living with HIV globally [3]. It is worth noting that over eighty percent of individuals infected with HIV-1 gain the virus via mucosal exposure [4]. As the pandemic continues to progress, the generation of a vaccine to combat this virus has become a top priority for researchers. However, the substantial genetic diversity of HIV gives rise to various genetic subtypes, constituting challenges for vaccine development [5]. The surface glycoproteins gp120 and gp41, present in the envelope (Env) of the virus, display excessive genetic variability. These glycoproteins are crucial in promoting the attachment of the virus into the T cells of the host [6]. Gp120 is extremely significant in the process of virus infection as it brings about the identification of specific receptors, including DC-SIGN, heparan-sulfate proteoglycans, and CD4 on the T cells of the host [7]. One strategy is to avoid viral attacks into CD4+ cells and thereby prevent infection by blocking the formation of the gp120-CD4 complex [8]. Furthermore, the production of neutralizing antibodies has garnered significant attention as they have the potential to bind to the junction of the external and internal domains of the gp120-CD4 binding site to rival CD4 receptors [9]. Different experiments have found that neutralizing antibodies can effectively inhibit the activity of gp120, making this glycoprotein a promising candidate for the development of attentive and potent HIV vaccines [4]. A subgroup of individuals living with HIV, indicated as elite controllers, acquire the incredible potential to remain asymptomatic and sustain high CD4+ cell counts for prolonged periods without the need for antiretroviral therapy (ART) [10]. These elite controllers have excesses of CD4+ and CD8+ cells, according to the analysis, that secrete IFN-γ, a cytokine that induces Th1 immune response, recommending its role in controlling HIV infection [11]. Moreover, the stimulation of CD4+ and CD8+ cells has shown effectiveness in combating HIV infection [12]. However, conventional strategies for designing and developing HIV vaccines have thus far been unsuccessful. As a unique and promising strategy, the therapeutic vaccination of individuals already infected with HIV-1 has appeared to prevent disease progression to AIDS [13].

A therapeutic vaccine against HIV-1 is meant to activate more effective and broader immune responses, especially targeting conserved viral epitopes, compared to the immune responses developed during natural infection. Therefore, the development of victorious HIV-1 therapeutic vaccine candidates has faced challenges, principally due to incompetent delivery systems or the suboptimal design of immunogens [13]. One promising strategy for the development of HIV-1 therapeutic vaccines involves the design and assemblage of artificial multiepitope immunogens. These immunogens are designed by choosing from prevalent viral antigens and a diverse array of immunostimulatory, protective, and T-cell epitopes. The objective is to provoke effective immune feedback against HIV-1 infection [14]. By employing this strategy, scientists hope to overcome earlier limitations and boost the effectiveness of therapeutic vaccines against HIV-1. Accordingly, in silico approaches that assist the discovery of potentially immunogenic peptides, known as epitopes, from the linear protein sequence are employed. Moreover, docking and molecular dynamics (MD) simulations can distinguish between different epitopes by assessing disparities in affinity and the stability of the peptide–protein complex on the major histocompatibility complex (MHC), including both MHC-I and MHC-II [15]. To analyze the dendrimer-G4-PAMAM-peptide complexes, Rodrguez-Fonseca and colleagues used 3D models of HIV-1 gp120. Female BALB/c mice received these complexes intravenously, either as individual peptides or as complexes. The research revealed that the immune response to the peptides was triggered at both the systemic and mucosal levels and that dendrimer–peptide complexes produced better IgG and IgA responses in serum and nasal washes [16]. Due to the significant role played by gp120 in host attachment and pathogenicity, we employed it as a primary antigen in the development of an HIV-1 vaccine. The focus of our study was on eliciting robust T-cell responses, specifically targeting the CTL and HTL. We present an effective in silico design strategy for designing a recombinant vaccine. To achieve this, we examined the conserved epitopes of gp120 and carefully selected suitable CTL and HTL epitopes capable of eliciting B lymphocyte responses against the gp120 protein. Subsequently, we designed the vaccine construct incorporating appropriate linkers and the Gb-1 adjuvant to enhance the induction of cellular immunity.

## 2. Results

HIV-1 envelope surface glycoprotein (Env) gp120 was the main target of the vaccine’s development. Specifically, from group M subtype B, which is the most frequent subtype in developed nations like the United States and Europe, we found 115 full sequences for the HIV-1 gp120 protein. It is also the most widely spread variant. We used a multiple alignment sequence analysis to obtain a consensus sequence to examine these protein sequences. VaxiJen v2.0 was employed to analyze the antigenicity of the acquired protein sequences. The number of transmembrane helices in the proteins was analyzed using the TMHMM v2.0 tool, and it was found to be between 0 and 1. Furthermore, the proteins were assessed for non-allergenic properties using the AllergenFP v1.0 tool.

To evaluate the antigenicity of the vaccine construct and assess other physiochemical characteristics, we determined the pI, half-life, and GRAVY scores [17]. The theoretical pI represents the pH at which a protein remains uncharged. The protein structure exhibited high aliphatic indexes, which indicate the relative volume of aliphatic amino acids in the protein’s side chain. The envelope glycoprotein gp120 displayed the highest extinction coefficient (72,515 *M^21^ cm^21^*) and appeared to be a hydrophilic protein with the lowest GRAVY score of −0.401. The GRAVY score evaluates the hydrophilicity or hydrophobicity of a compound. A negative GRAVY score indicates hydrophilic properties, suggesting good solubility in water, while a positive GRAVY score suggests insolubility. Subsequently, the consensus sequence was analyzed using epitope predictors, resulting in the identification of different peptides. Table 1 presents all the immunoinformatic software used in the current experiment.

### 2.1. Linear and Conformational B Cell Epitope Prediction

**Figure 1:**
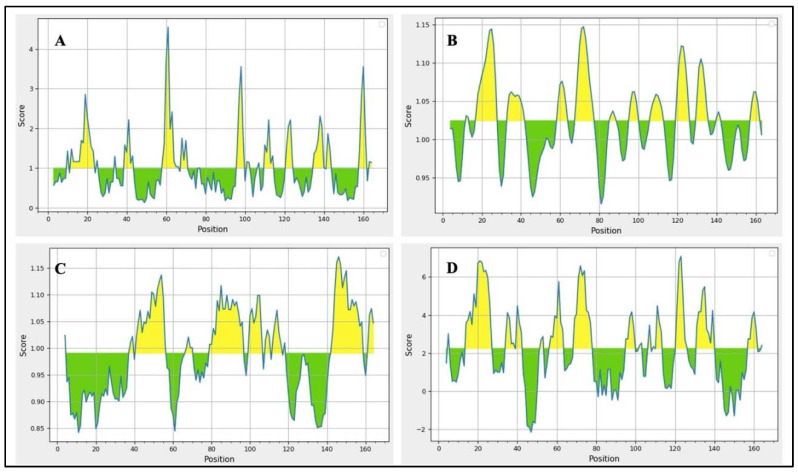
B-cell epitopes were identified using various IEDB epitope servers. Epitopes exhibiting positive interactions are highlighted in yellow. (**A**) The surface accessibility of B-cell epitopes was assessed using the Emini tool. (**B**) The flexibility of selected epitopes was evaluated using the Karplus & Schulz tool. (**C**) The antigenic potential of the final epitopes was observed using the Kolaskar & Tongaonkar method. (**D**) The hydrophilicity of epitopes was examined using the Parker method. The residues with scores higher than the threshold were predicted to be part of an epitope, as indicated in yellow.

**Figure 2:**
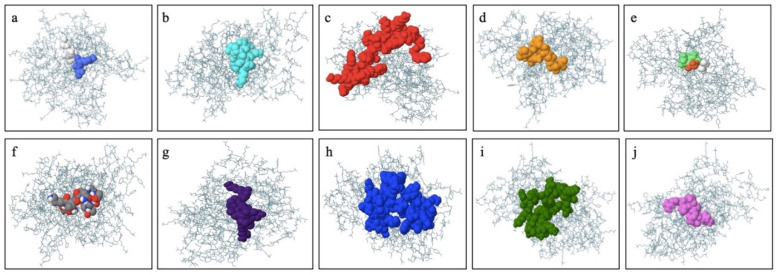
The final vaccine construct displays a three-dimensional representation of conformational or discontinuous epitopes (**a**–**j**) found within the highest antigenic polyprotein of gp120. The epitopes are depicted on the surface using various colors, while the bulk of the polyprotein is represented as sky blue sticks.

IEDB, BepiPred 2.0, and iBCE-EL were employed to analyze linear B-cell epitopes within the final construct. The initial prediction was performed using IEDB and BepiPred 2.0, while the iBCE-EL tool was employed to verify the predictions made by BepiPred [18]. If a B-cell epitope analyzed using iBCE-EL was not identified via any of the other three servers, it was discarded. To ensure the B-cell epitopes’ efficacy, they needed to be situated in solvent-exposed regions of the antigens, enabling their interaction with B cells. Thus, predicting the surface accessibility of the structural protein sequence was crucial. In this study, the Emini tool was employed to assess the surface accessibility of the HIV-1 gp120 protein sequence (Figure 1), and epitopes that were not exposed on the surface were excluded. Overall, 11 potential linear B-cell epitopes were identified from the selected proteins (Table 2). The flexibility, antigenicity, and hydrophilicity of the designed epitopes were predicted using the Karplus & Schulz, Kolaskar & Tongaonkar, and Parker methods, respectively (Figure 1). It is important to note that the unique protein’s structure and folding may give rise to unique conformational B-cell epitopes, which required further evaluation. The ElliPro tool was utilized to analyze conformational B-cell epitopes in the refined 3D structure. As a result, 11 new conformational B-cell epitopes were evaluated using ElliPro, involving 315 residues and scoring between 0.525 and 0.786. A three-dimensional model structure of confirmational epitopes in the final vaccine construct is shown in Figure 2.

### 2.2. Selection of CTL Epitope Prediction

CTL plays a key role in identifying infected cells by utilizing MHC class I molecules to bind with specific CTL epitopes. To predict MHC class I binding epitopes within gp120, the IEDB analysis tool was employed. Overall, 48 predicted 8- to 12-mer peptide CTL epitopes were determined from the selected protein based on their high immunogenicity and antigenicity (Table 3). Furthermore, the binding affinity of the selected epitopes to mouse MHC-I alleles was evaluated. The epitope candidates underwent screening based on their calculated MHC-I processing mean values, immunogenicity values, and population coverage across different geographic areas worldwide. The epitopes with the highest scores were further evaluated for their degree of conservancy among various HIV-1 clades, as well as their allergenicity, toxicity, and hemotoxicity. Moreover, the MHC-I epitopes were selected based on a higher binding value according to the IEDB tool. Any selected MHC-I epitopes that overlapped with one another were excluded from the final epitope selection process.

### 2.3. CTL Immunogenicity Prediction

The processing of T-cell epitopes and the immunogenicity scores of the selected MHC-I epitopes were predicted using the IEDB server and the European Molecular Biology Open Software Suite (EMBOSS 6.4.0). Upon analysis, it was found that the selected epitopes had the highest immunogenicity values according to the IEDB immunogenicity tool. The algorithm used for prediction considered both immunogenicity and antigenic scores to determine the epitopes with the highest potential for immune response.

### 2.4. Selection of HTL Epitopes Prediction

HTLs are essential for the function of other immune cells, and they identify infections by binding with certain HTL epitopes on MHC class II molecules. It was uploaded to the IEDB server to assess the binding of peptides to MHC class II alleles found in the HIV-1 gp120 antigen. As a result, the chosen protein yielded a total of 46 possible HTL epitopes (Table 4). The predicted peptides, which were high-potential HTL epitopes because they had high binding affinity values for a considerable number of HLA-II alleles, ranged in length from 14 to 16 amino acids. The HTL epitope candidates were then subjected to screening based on their estimated population coverage across several global geographic areas. Therefore, the best-scoring epitopes were analyzed for their allergenicity, toxicity, hemotoxicity, capacity to stimulate cytokine production, and degree of conservation among various HIV-1 clades. Epitopes from the MHC class II that displayed overlap were regarded as separate epitopes. To decide which MHC class II epitopes would be included in for the final construct, all MHC class II epitopes were examined for their capacity to stimulate the production of IFN-γ.

### 2.5. Selection of Most Promising Epitopes

To develop the vaccine, T-cell and B-cell epitopes were identified and then evaluated based on their physiochemical characteristics. CTLs can recognize antigens, whereas HTLs are involved in the stimulation of other immune cells, such as B cells, macrophages, and CTLs [19]. B cells can change into plasma cells, which make antibodies, thus mediating the humoral immune response. However, the humoral immune feedback weakens with time and is not as potent as the cell-mediated immune feedback [20]. Meanwhile, the cell-mediated immune feedback, which releases antiviral cytokines and selectively identifies and destroys infected cells, offers stronger and longer-lasting protection. The HIV-1 envelope glycoprotein (Env) gp120 was used in this investigation as the model for identifying epitopes. The server generated a large number of epitopes; the most promising T-cell epitopes were chosen for additional examination. B-cell epitopes longer than seven amino acids were also selected for additional screening. The T-cell and B-cell epitopes were picked after being analyzed using several parameters, including high antigenicity, non-allergenic and non-toxic qualities, conservation among the strains, and divergence from the human proteome. The vaccine was finally designed using epitopes that satisfied these requirements. The HTL epitopes’ capacity to elicit cytokines was evaluated, and only those that had the potential to do so were used in the development of the vaccine. The promising epitopes selected for use in the final vaccine design are shown in Table 5.

### 2.6. Proteasomal Cleavage/TAP Transport

The identification of the gp120 antigen employing the NetCTL1.2 servers was warranted given the importance of proteasomal cleavage and TAP transport in the antigen presentation pathway. Each of the CTL epitopes expressed scores for tap transport and proteasomal cleavage are shown in Table 3. For the construction of the final vaccine, the CTL epitopes with the greatest immunogenicity values were chosen.

### 2.7. IFN-γ Inducing Epitopes

A key function in antiviral defense systems is played by the cytokine IFN-γ. By triggering macrophages and natural killer cells, it increases both innate and adaptive immune feedback. IFN-γ also improves the way that MHC molecules react to antigens. The final HTL epitope selection was based on the HTL epitopes’ capacity to stimulate IFN-γ production and their affinity for binding to MHC Class II, which is necessary for activating T-helper cells. Many epitopes were eliminated during the screening process due to the IFN-γ filter, as they were predicted to have a negative IFN-γ release. Table 4 displays the 46 HTL epitopes that had positive predictions for both IFN-γ induction and immunogenicity. Similarly, any epitopes that were predicted to have a negative IFN release but did not appear in the group of 46 epitopes were excluded.

### 2.8. Population Coverage Analysis

**Figure 3:**
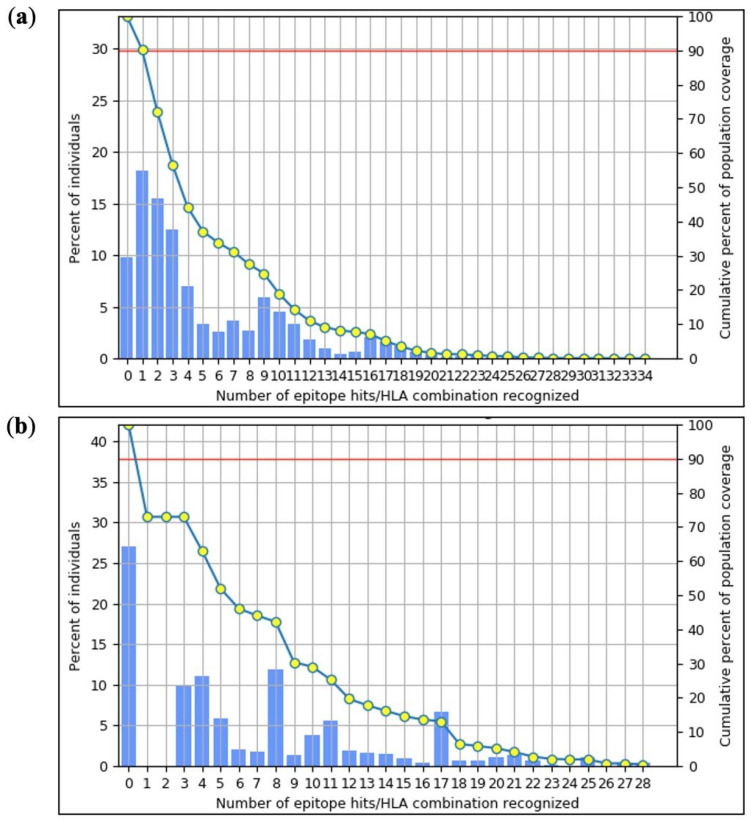
The population coverage (%) related to the selected epitopes’ HLA binding alleles was considered, taking into account both worldwide and average percentages. (**a**) For MHC class I restricted alleles, the selected epitopes represent a population coverage of 90.23% worldwide. (**b**) For MHC class II restricted alleles, the selected epitopes represent a population coverage of 72.95% worldwide. In the graph, the line (-o-) illustrates the cumulative percentage of population coverage for epitopes, while the bars depict the coverage for each individual epitope.

The IEDB population coverage server was employed to assess the extent of population coverage for the selected epitopes. The results of the population coverage scanning revealed that the epitopes associated with MHC class I alleles enclosed approximately 90.23% of the global population, while the epitopes linked to MHC class II alleles enclosed about 72.95% of the world population. As depicted in Figure 3, the highest population coverage was observed in both Class I and II epitopes of the gp120 antigen. When combined, these epitopes from the two MHC classes accounted for an average population coverage of 95.38%, indicating a very high likelihood of effectiveness in any region of the world. The population coverage analysis indicates that a significant portion of the global population acquired the target MHC alleles. Consequently, we anticipate that our constructed vaccine would successfully thwart the virus on a global scale.

### 2.9. Antigenicity, Allergenicity, and Solubility Assessment

The antigenicity of each of the B-cell, CTL, and HTL epitopes was assessed using the Vaxijen 2.0 and AntigenPro servers. Epitopes that exhibited potential antigenic values were chosen for the final vaccine design. The allergenicity of each type of epitopes was predicted using the AllergenFP 1.0 and AllerTOP 2.0 servers. Only non-allergenic epitopes were selected for the final vaccine formulation. The solubility of the epitopes was evaluated using the SolPro and Protein-sol tools. The predicted solubility values aided in the finalization of epitopes based on their favorable solubility characteristics. The results pertaining to different immunogenic parameters for each epitope mentioned in Table 2, Table 3 and Table 4.

### 2.10. Toxicity and Physicochemical Properties Assessment

It is pivotal to evaluate the absence of toxicity potential in the vaccine, as well as evaluate its physicochemical properties, to understand its interaction with the environment [21]. To predict toxicity, we utilize the ToxinPred server. Additionally, the ExPASy ProtParam Tool helped us to predict various physicochemical properties such as hydropathicity, charge, half-life, the instability index, pI, and molecule weight. To examine the sequences of B-cell, CTL, and HTL epitopes, we assessed their overlapping peptides with a length not exceeding 20 amino acids using the ToxinPred server. After identifying the non-toxic epitopes, they were included in the final vaccine construct based on various evaluation tests. Subsequently, the complete vaccine sequence, including the adjuvant, underwent analysis to ensure the absence of any toxic peptides. The remaining subunits and the 6xHis tag were evaluated using the SVM prediction mode in the ToxinPred tool, confirming their non-toxic nature. Additionally, the hydropathicity value of each epitope was predicted, and only those epitopes with negative values were considered. The negative value indicates that our epitopes are hydrophilic and can readily interact with water molecules. The results pertaining to the toxicity and physiochemical parameters of each epitope are shown in Table 2, Table 3 and Table 4.

### 2.11. Epitopes MHC Restriction and Cluster Assessment

**Figure 4:**
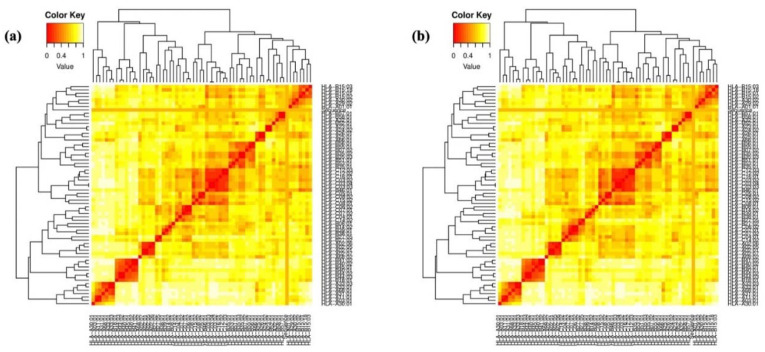
Cluster analysis was performed on the HLA alleles for both MHC molecules, represented through a heat map. (**a**) The cluster of the MHC-I (VTVYYGVPVW, RAKWNNTLK, SVNFTDNAK, APTKAKRRVV, FNSTWFNSTW, GVAPTKAKR, KVQKEYAFFY, QKEYAFFYKL, TIGKIGNMR, and WQKVGKAMY) epitopes was represented. (**b**) The cluster of the MHC-II molecules (EIKNCSFNISTSIRG, EPLGVAPTKAKRRVV, FYKLDIIPIDNDTTS, GKVQKEYAFFYKLDI, INCTRPNNNTRKRIR, and GFAILKCNNKTFNGT) epitopes was represented.

The selected epitopes underwent validation for MHC restriction analysis via the MHC cluster after the physiochemical study. Following a cluster analysis to assess the interacting alleles, the results were shown as a heat map for MHC class I and MHC class II (Figure 4). The epitopes were clustered based on their correlation with HLA. Strong interactions are shown by the color red, while weak interactions are shown by the color yellow.

### 2.12. Multiepitope Subunit Vaccine Modeling

**Figure 5:**
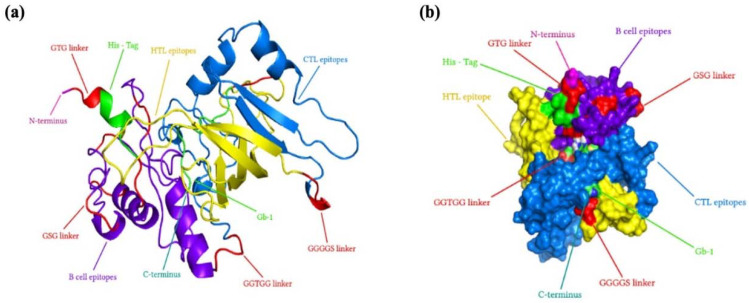
The sequences, locations, and representations of the immunodominant B-cell, CTL, and HTL epitopes in the final vaccine construct, as well as the 3D view and locations of the immunodominant epitopes of the gp120 monomer, are shown. (**a**) The sequences and positions of the immunodominant epitopes are depicted in a cartoon shape. (**b**) The immunodominant epitopes are represented in a 3D view using the PyMOL 2.3.4 program. The surface representation shows the locations of the immunodominant epitopes on the closed conformation of the 3D gp120 protein of HIV-1.

**Figure 6:**
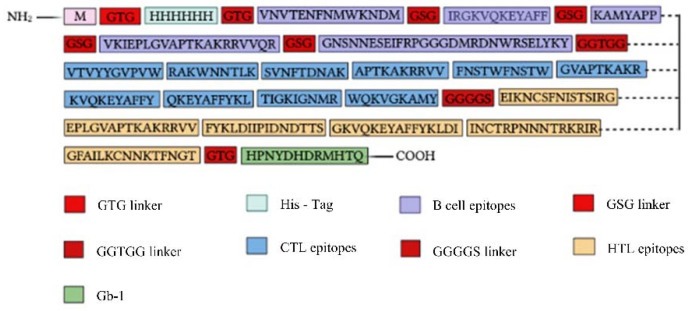
Representation of the HIV-1 gp120 construct schematic diagram, including B-cell, CTL, and HTL epitopes, linkers, and adjuvants. The Histidine-Tag is connected to the B-cell epitopes through a GTG linker. The B-cell epitopes are connected to the CTL epitopes using GGTGG linkers. The CTL epitopes, in turn, are connected to the HTL epitopes using GGGGS linkers. Finally, the HTL epitopes are connected to the adjuvant through a GTG linker.

As explained in “Selecting the most promising epitopes,” the epitopes with high antigenicity, an absence of allergies and toxicity, conservation across selected strains of gp120, the potential to provoke cytokines specifically for HTL epitopes, and non-similarity to the human proteome were identified as the most favorable epitopes. These epitopes were selected for the development of the vaccine. Mostly subunit vaccines typically possess an adjuvant to effectively activate innate immunity and induce an immune response. Given that antimicrobial peptides have shown potential in stimulating both innate and adaptive immune responses [22], our laboratory’s recently discovered Gb-1 was selected as a suitable adjuvant for the vaccine. In the process of constructing the multiple-epitope vaccine in this study, a total of 5 B cell epitopes, 10 CTL epitopes, and 6 HTL epitopes were selected according to various immunogenic tests. The 3D view of the final immunodominant epitopes is shown in Figure 5. The final selected epitopes were linked together using specific linkers. The GTG linker was employed to link the start codon with the Histidine Tag, positioned at the N-terminal of the vaccine design. GSG linkers were utilized to bind B-cell epitopes, while the GGTGG linker was employed between the end of B-cell epitopes and the beginning of CTL epitopes. For connecting HTL epitopes with CTL epitopes, a GGGGS linker was used. Gb-1, serving as the adjuvant, was connected using GTG linkers at the C-terminal site. A 315-amino-acid residue vaccine design was constructed after proper combination and randomization. The final vaccine sequence is depicted in Figure 6.

### 2.13. Antigenicity, Allergenicity, and Physicochemical Composition of Vaccine Construct

During the antigenicity and allergenicity tests, the HIV-1 gp120 vaccine showed high antigenicity and non-allergenicity, demonstrating its ability to elicit the necessary immune response without triggering unfavorable allergy responses. The formulated gp120 construct’s physicochemical properties were then assessed, confirming its appropriateness. The vaccine’s molecular weight was found to be 35,493.37 Da, and its chemical formula was found to be C_1587_H_2443_N_465_O_444_S_11_. The aliphatic index (AI), a thermostability metric, was found to be 53.52, indicating that the vaccine is still stable at the average body temperature of an adult. The vaccine’s theoretical isoelectric point (PI) of 10.13 demonstrated that it had an alkaline nature. The negative GRAVY score (−0.741) of the vaccine proteins expressed that they were hydrophilic. The gp120 vaccine was anticipated to have a half-life of more than 10 h in the *E. coli* cell culture system and 30 h in mammalian reticulocytes, indicating the possibility for easy mass manufacturing and purification using the *E. coli* system. High solubility was also demonstrated by the HIV-1 gp120 vaccine upon overexpression in *E. coli* cells. Determining the post-production processing of the vaccine depends on the ability to estimate solubility in *E. coli*. If the recombinant protein is insoluble, incorrect folding or the development of insoluble inclusions in the human body may render it nonfunctional. Additionally, vaccine solubility makes vaccine purification during post-production processing easier [23]. Collectively, these results show promise for using the projected vaccine as an efficient HIV-1 prevention method. Table 6 represents an overview of the physiochemical findings for the final vaccine design.

### 2.14. Prediction of the Secondary Structure of the Vaccine Construct

The distributions of amino acids’ α-helix, β-strand, and coil structures in the vaccine protein were examined using three prediction methods: PSI-blast-based secondary structure PREDiction (PSIPRED), the Self-Optimized Prediction Method With Alignment (SOPMA), and Phyre2 servers.

**Figure 7:**
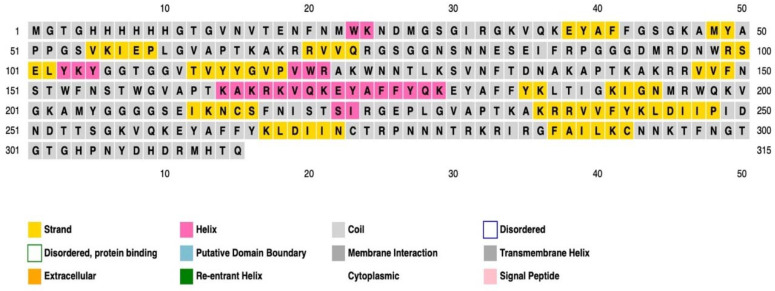
Graphical representation of the secondary structure features of the final vaccine construct. The alpha helix residues are in pink, the beta strand residues are in yellow, and the coil residues are in grey. Upon analyzing the predicted secondary structure, it is revealed that the final vaccine comprises 10.2% alpha helix, 22.6% beta strand, and 60.2% coil.

**Figure 8:**
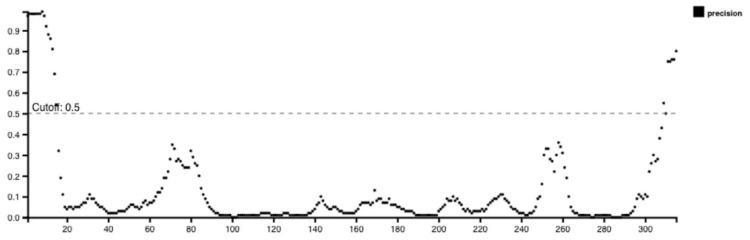
Disorder regions in the secondary structure of the designed vaccine predicted via the PSIPRED Tool. This tool has provided valuable insights into the secondary structure of the designed vaccine. In particular, it has identified disorder regions within the protein sequence. According to the analysis, amino acids in the input sequence are considered disordered when the dotted line exceeds the cutoff value of 0.5, representing the confidence threshold.

The findings demonstrated obvious deviations in the proportions of amino acids in the highest and lowest populated structures while showing consistent outcomes across all four servers for each of the α-helix, β-strand, and coil structures. The PSIPRED prediction tool was the main topic of our final evaluation. As given in Figure 7, shows a visual depiction of the characteristics of the secondary structure. The final vaccine built, as evaluated by the SOPMA server, was 23.65% α-helix, 33.2% β-strand, and 45.08% random coils, as opposed to the 26.32% α-helix, 39% β-strand, and 40.74% random coils anticipated by the Phyre2 server. According to the secondary structure study, 14% of the areas were labeled as disorder regions (Figure 8).

### 2.15. Modeling of Three-Dimensional (3D) Vaccine Construct, Refinement, and Validation

**Figure 9:**
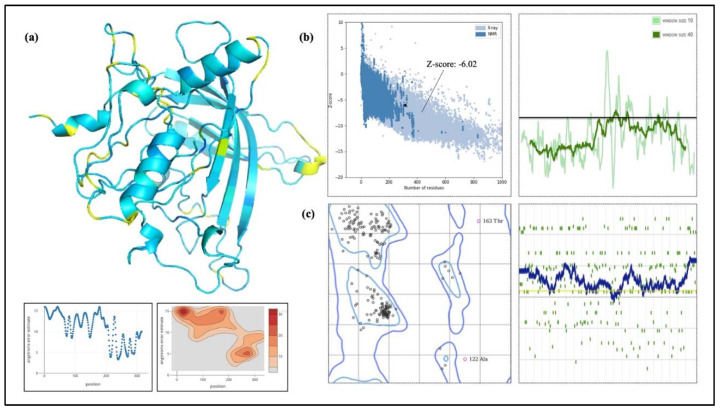
Representation and Validation of the HIV-1 gp120 vaccine using in silico tools. (**a**) The HIV-1 gp120 vaccine construct was modeled in 3D, employing refined parameters through Galaxy Refine. (**b**) Prosa-web generated the Z-score and local model quality energy score, providing validation for our 3D vaccine construct. (**c**) The Ramachandran plot, associated with the vaccine’s 3D structure post-refinement, further validated the accuracy of our vaccine design. Additionally, the 3D prediction was verified through Verify 3D, indicating that the vaccine structure achieved a remarkable 92.70% residue score.

RoseTTAFold was employed to model the HIV-1 gp120 3D structure, which was then improved to achieve a greater similarity to the native or natural structure of the protein. The vaccine design’s projected 3D structure is shown in the Figure 9. The GalaxyRefine tool was employed to enhance the final vaccine’s 3D structural model. Based on its higher model quality ratings, model 1, among the five refined models produced by GalaxyRefine, was chosen as our final vaccination model. The Global Distance Test-High Accuracy (GDT-HA) score between this improved model and the original model was found to be 0.9595, showing an increased level of similarity between the two protein structures. For this model, the Root Mean Square Deviation (RMSD) value, which is a measure of the separation between atoms in the structure, was 0.403. An RMSD value between 0 and 1.2 is often considered significant, and a lower RMSD number denotes more stability. Consequently, a stable protein structure can be shown in this model. The MolProbity value, which measures the model’s crystallographic resolution, was found to be 2.108 for the vaccination model that we uncovered. This score is much lower than that of the original model, indicating that the 3D model’s key flaws were effectively eliminated throughout the refining process. The model’s refinement clash value, which counts the amount of unfavorable all-atom steric overlaps, is 14.1, which indicates a significant increase in model stability. The Ramachandran plot value, which showed the size of energetically favored areas, was improved via refining from 92.0% to 93.9%. The Ramachandran plot value is often accepted at a value greater than 85%. ProSA-web was used to validate the overall model quality of the final vaccine design after it had been improved. ProSA forecasts a Z-score of −6.02 for the improved model, which is within the acceptable range for native proteins of comparable size (Figure 9). Overall, this suggests a high level of model quality. The Z-Score is frequently used to verify proteins that have been modeled using NMR or X-ray techniques. Proteins with less than 200 amino acids are normally studied using the NMR approach, whereas proteins with more than 200 amino acids are studied using the X-ray technique. ProSA also evaluates the local model quality and shows the residue values. Negative values indicate that there is no mistake in the model structure. The Ramachandran graph was used to analyze the predicted model, and it was discovered that 93.9% (294/313) of all residues were located in favored regions (98%). The angles of phi and psi are used to organize amino acids in the Ramachandran graph (Figure 9).

### 2.16. Disulfide Bond Engineering of the Designed Vaccine

**Figure 10:**
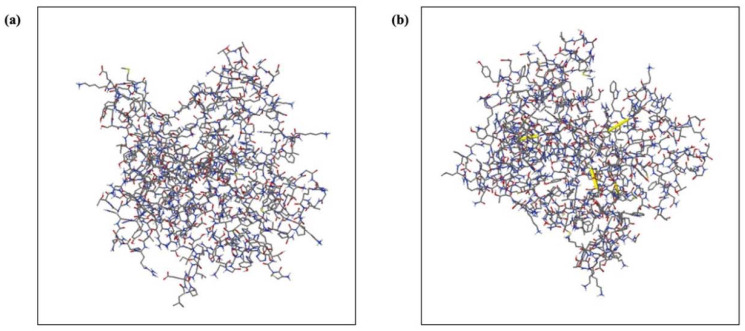
Stability of the vaccine construct through disulfide bond engineering. (**a**) The original form of the vaccine construct is represented without any mutations. (**b**) In the mutant form of the vaccine construct, six pairs of amino acids, including Gly11-Gln73, Ala122-Asn125, Trp153-Asp308, and Lys202-Tyr205, are depicted in yellow sticks. These specific amino acid pairs have undergone modifications to incorporate disulfide bonds.

The Disulfide by Design v2.12 web server found a total of 22 pairs of amino acid residues eligible for inducing disulfide mutations during the disulfide engineering procedure. These pairs were carefully evaluated based on their χ3 angle and energy value. Ultimately, only four pairs of amino acids met the required criteria: the χ3 angle had to fall within the range of −87 to +97°, and the energy score had to be below 2.2 kcal/mol. Consequently, mutations were introduced in six residue pairs, including Gly11-Gln73, Ala122-Asn125, Trp153-Asp308, and Lys202-Tyr205. The χ3 angles for these mutations were +111.82, −83.21, −89.79, and +115.80°, while their energy values were 1.23, 1.34, 1.09, and 2.22 kcal/mol, respectively (Figure 10).

### 2.17. Molecular Docking of the Vaccine Construct with TLRs

**Figure 11:**
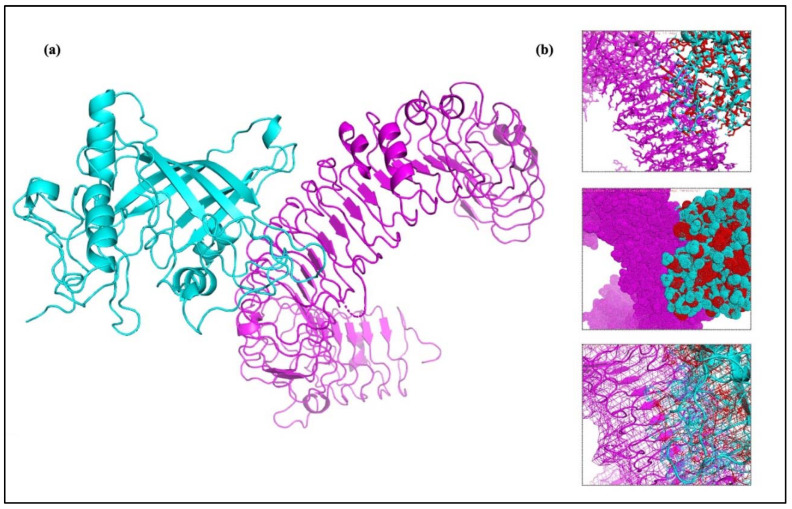
Molecular docking of the TLR-2 complex and vaccine construct. (**a**) A cartoon representation of the Vaccine TLR-complex is depicted, with the TLR component shown in a vibrant magenta color, while the vaccine complex is represented in a striking cyan color. (**b**) Multiple interaction views of the TLR-2 and vaccine complex are generated using PyMOL. The interaction residues of the TLR-2 and vaccine complex are displayed in distinct colors, highlighting their respective roles in the binding interface.

**Figure 12:**
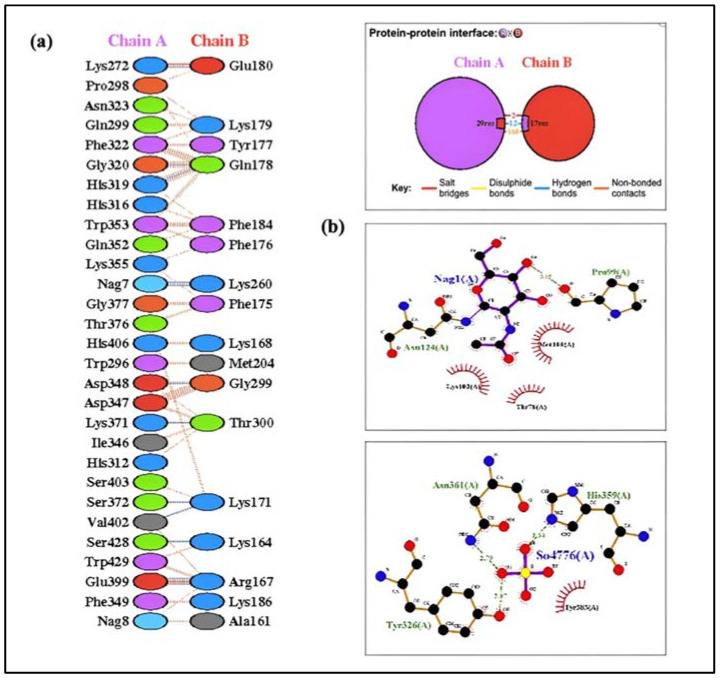
This figure illustrates the binding interaction between the active residues of a docked complex involving human TLR-2 and a vaccine construct. (**a**) In this complex, chain A represents the active residues of TLR-2, while chain B represents the active residues of the vaccine construct. (**b**) The docked conformation and interaction of TLR-2 and the vaccine construct are depicted via Ligplot analysis, highlighting the presence of hydrogen bonding and hydrophobic interactions between the construct.

**Figure 13:**
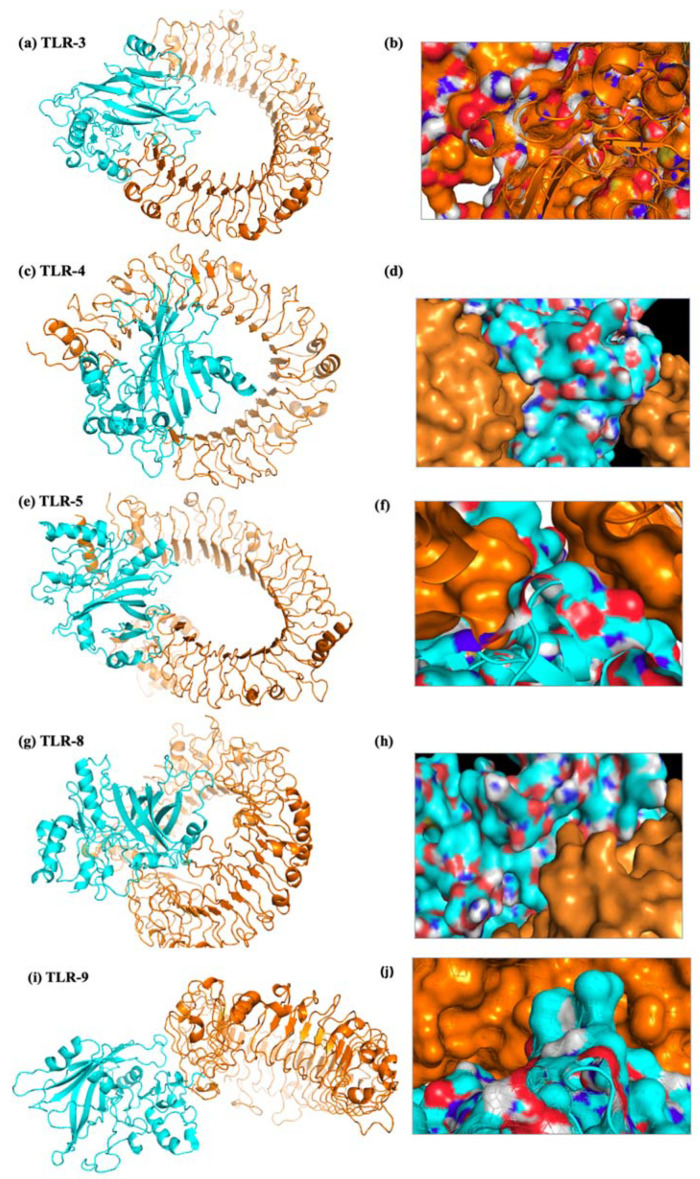
Molecular docking analysis was conducted to investigate the interaction between the HIV-1 gp120 vaccine construct and human toll-like receptors (TLRs). Cluspro was utilized to identify the docking regions between the vaccine construct and the TLRs. The figure displays the docking interaction between vaccine construct and TLRs, with a closer view of the interaction. (**b**) represents a closer view of TLR-3, while (**d**) depicts a closer view of TLR-4. Similarly, (**f**) illustrates a closer view of TLR-5, (**h**) portrays TLR-8 in closer detail, and (**j**) presents a closer view of TLR-9.

Understanding the relationship between the immune cell and the vaccine design is important for producing a reliable and consistent immune response. Molecular docking serves as a useful approach for evaluating the stability and binding affinity of a docked complex made up of a ligand and a receptor molecule. The interaction between vaccines and TLRs is a key indicator of effective protection against infections because TLRs play an effective role in triggering a healthy immune response. The final vaccine construct’s molecular docking mechanism used TLR-2, TLR-3, TLR-4, TLR-5, TLR-8, and TLR-9. Additionally, the adjuvant Gb-1 sequence was included at the C-terminal of the final construct. To ensure accurate predictions, docking was performed using two different servers. The findings of the docking experiment indicated that the vaccine design exhibited robust and satisfactory interactions with the TLRs across all utilized servers. The HADDOCK and ClusPro 2.0 servers were employed for molecular docking between the refined 3D structure of the final vaccine and the TLRs immune receptor. The docking interaction between the HIV-1 gp120 vaccine and TLRs was analyzed using the HADDOCK server. The complexes with the highest rankings were chosen based on the whole-molecular HIV-1 gp120-protein complexes from HADDOCK, considering the lowest mean RMSD. Additionally, an examination of the HADDOCK server output revealed the significance of electrostatic bonds in the interactions observed in the HIV-1 gp120-TLR-2 complex (Figure 11). The HADDOCK results were subjected to further verification using the Pdmsum and LigPlot analysis tools, which confirmed the selection of the best docking results (Figure 12). The complex with the lowest energy value was selected as the best-docked complex through ClusPro docking analysis. This indicated that the vaccine effectively occupied the receptor and displayed strong binding affinity. The lowest energy levels obtained for the docking of the HIV-1 gp120 construct with TLR-2, TLR-3, TLR-4, TLR-5, TLR-8, and TLR-9 were −789.0, −966.6, −882.8, −1251.0, −900.9, and −1047.0, respectively (Figure 13). The multiple-epitope peptide constructions and TLRs in the docked complexes have excellent binding affinity, as shown by their low energy levels.

### 2.18. Molecular Dynamics Simulation

**Figure 14:**
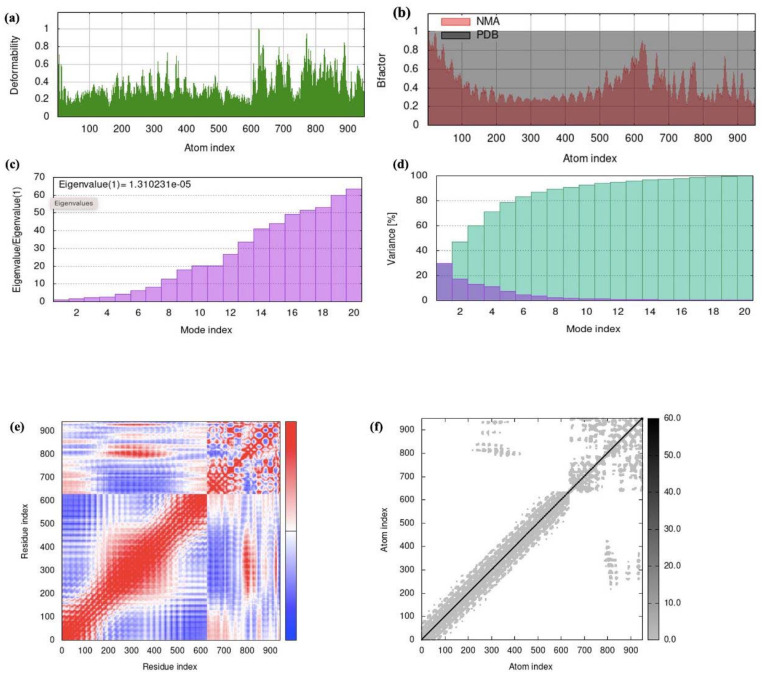
The vaccine−TLR-2 docked complex underwent molecular dynamics simulation. (**a**) A main-chain deformability simulation was performed, identifying regions with high deformability known as hinges. (**b**) B-factor values were calculated using normal mode analysis, providing a measure of uncertainty for each atom. (**c**) The eigenvalue of the docked complex was determined, indicating the energy required to deform the structure. (**d**) The covariance matrix between pairs of residues was analyzed, with red indicating correlation, white indicating no correlation, and blue indicating anti-correlation. (**e**,**f**) An elastic network model was generated to visualize the connections between atoms and springs. The springs are more rigid if their shades of grey are darker.

We used the iMOD web tool to evaluate the stability and motions of the docked complex between the vaccine and TLR-2. The main-chain deformability reveals hinges with significant deformability (Figure 14a). The β-factor scores are determined via normal mode analysis, which is proportional to the root mean square and represents the unreliability surrounding each atom (Figure 14b). The eigenvalues are closely linked to the energy required for structural deformation, with the eigenvalue of the complex measuring 1.310231 × 10^−5^ (Figure 14c). The covariance matrix represents the correlations between pairs of residues (red: correlated; white: uncorrelated; blue: anti-correlated) (Figure 14d). Moreover, the elastic network model demonstrates the interconnectedness between atoms and springs within the system (Figure 14e,f). Based on the results of the molecular dynamics simulation, our vaccine model exhibits stability, as indicated based on the analysis conducted.

### 2.19. Codon Optimization and In Silico Cloning

**Figure 15:**
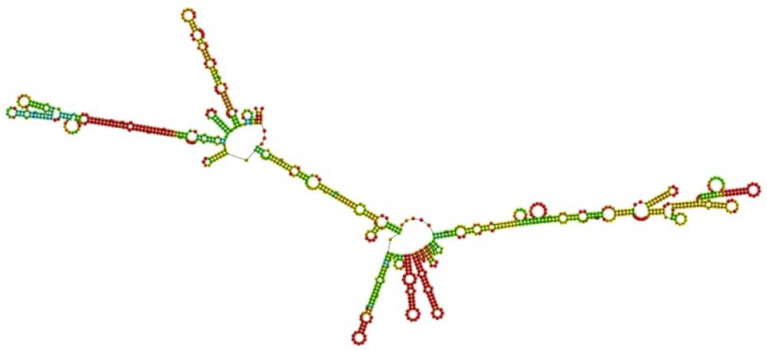
mRNA structure of the HIV-1 gp120 vaccine construct. The mRNA structure of the HIV-1 gp120 vaccine construct was determined using the RNAfold servers. The prediction yielded a minimal free energy score of −378.55 kcal/mol, indicating the stability of the vaccine design’s mRNA structure.

JCAT was employed to optimize the nucleotide sequences of the final vaccine design. To prevent bacterial ribosome binding sites, rho-independent transcription terminators, and restriction enzyme cleavage sites, JCAT’s characteristics were modified. For in silico cloning, the cDNA sequence produced via reverse transcription was used. *E. coli* K12 was chosen as the host expression organism. Analysis of the codon optimization for the HIV-1 gp120 construct revealed a 61.90% GC content. The CAI value showed the high expression potential of the selected gene, with a CAI value of 0.60 for HIV-1 gp120 in *E. coli* cells. Restriction enzymes *NcoI* and *XhoI* were linked to the N-terminal and C-terminal of the nucleotide sequence, respectively. A stop codon was also included after the Gb-1 sequence. The vaccine design mRNA structure was predicted using the RNAfold servers, which resulted in a minimal free energy score of −378.55 kcal/mol (Figure 15). The anticipated HIV-1 gp120 vaccine may remain stable after expression in vivo if the minimal free energy score is lower, which denotes more mRNA stability. Overall, the techniques used in this work suggest the possibility of using the gp120 vaccine as an accessible and low-cost strategy for preventing HIV-1 infection. However, to support the results of this study, more in vivo and in vitro research is needed.

### 2.20. Immune Simulations of Vaccine Construct

**Figure 16:**
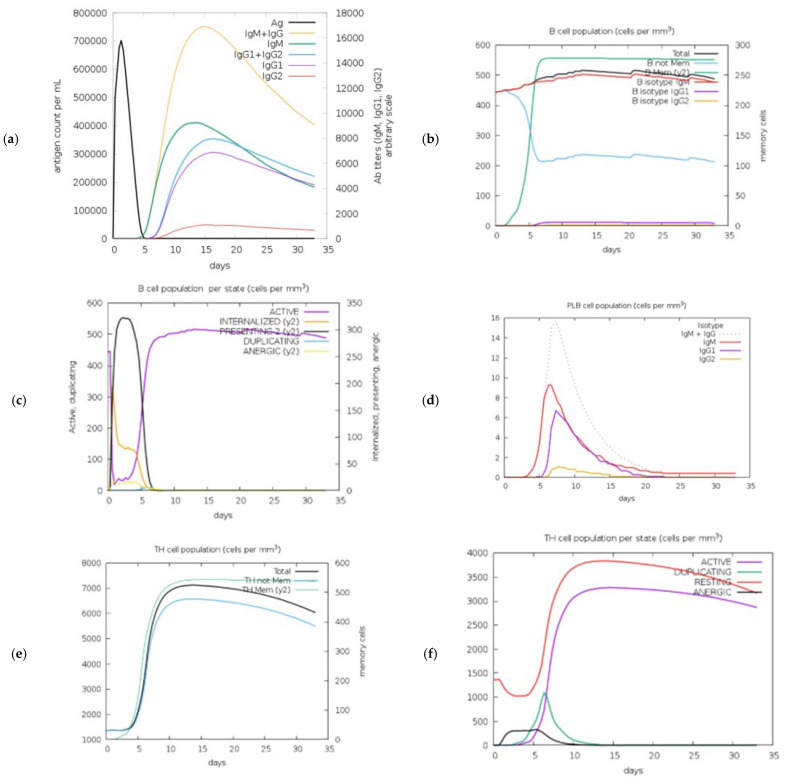

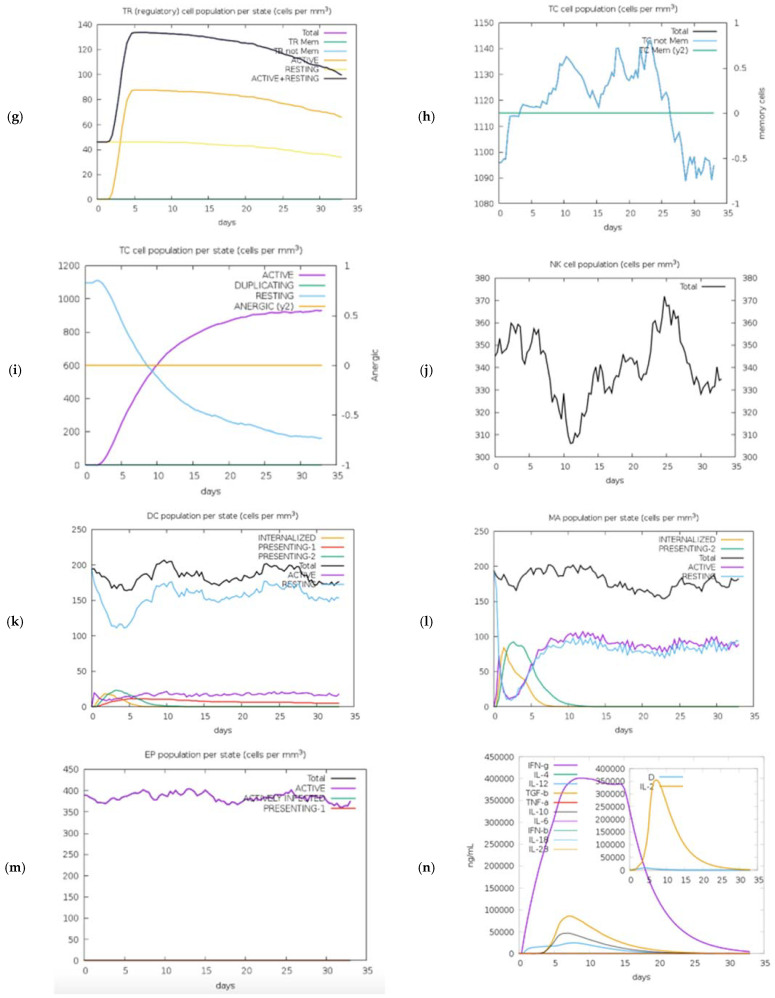
C−ImmSimm represents the immune stimulation of the best-predicted HIV-1 gp120 vaccine. (**a**) The immunoglobulin and immunocomplex responses to the HIV-1 gp120 vaccine inoculations are indicated by colored lines. (**b**–**h**) The number of plasma B cells, HTLs, and CTLs exhibited progressive increases, indicating the robust development of an immune response characterized by high potency, immunological memory, and efficient removal of antigens from the host. (**i**–**l**) The increase in DCs and macrophages exhibited greater antigen presentation by APCs, and the activation of helper T cells demonstrated the superior adaptive immunity of the vaccine. (**m**,**n**) The vaccine was shown to be capable of inducing the production of IFN-γ, IL-23, IL-10, IL-8 and IL-6, which are vital for triggering immune feedback and protecting the body against viruses.

The immunological stimulation of the HIV-1 gp120 vaccine was evaluated using the C-ImmSimm tool, which also forecasted the abilities of epitopes to induce adaptive immunity. The immunological interactions between the epitopes and their particular targets were also established through this investigation. Results from the C-ImmSim online server showed that secondary immune response generation increased and followed the real immunological response. According to the results of the immunological simulation study, the HIV-1 gp120 vaccine can produce an immune response that is characteristic of the body’s immune system. With gradual doses, the vaccine was assessed to produce strong primary immunological responses (Figure 16a). Moreover, the primary immune feedback gradually improved with each dosage, and the secondary immune feedback was elevated. Furthermore, there were progressive increases in the numbers of plasma B cells, HTLs, and CTLs, indicating the development of an immune response with high potency, immunological memory, and efficient antigen removal from the host (Figure 16b–h). While the increase in dendritic cells and macrophages exhibited greater antigen presentation by APCs, the activation of helper T cells demonstrated the vaccine’s superior adaptive immunity (Figure 16i–l). IFN-γ, interleukin-23 (IL-23), IL-10, and IFN-γ have all been shown to be capable of being produced by the vaccine, which is vital for triggering immune feedback and protecting the body against viruses (Figure 16m,n). APCs, cytokines, active B cells, and T cells were among the promising characteristics of the vaccine that the immune simulation studies indicated were produced in substantial quantities. This shows that after being administered to the host, the polyvalent HIV-1 gp120 vaccine produced good immunogenic reactions. These findings show that following each injection, substantial immunological memory development, enhanced antigen clearance, and significant secondary immune responses occur.

## 3. Discussion

The field of bioinformatics has emerged as a pivotal force in the realm of vaccine development, owing to its ability to predict immunogenic peptides that facilitate the development of vaccines that are both effective and safe. By utilizing peptide predictors, the costly and unwanted side effects associated with vaccines derived from attenuated pathogens, be they living or inert, can be minimized [24]. While remarkable progress has been made in combating AIDS through antiretroviral treatments, the urgent need for an effective HIV-1 vaccine remains paramount in our efforts to curb this perilous global epidemic [25]. Regrettably, most previously devised vaccines have fallen short of expectations, largely due to their limited potency against the promptly mutating nature of the virus, as they have predominantly targeted a narrow range of HIV genotypes [26]. According to studies using the in silico developed multiple-epitope EP HIV-1090 vaccine, these vaccines’ inability to elicit strong cellular and HTL feedback is another reason why they are ineffective at preventing HIV-1 [12], as well as for their failure to provoke broadly neutralizing antibodies, as demonstrated by experiments conducted on BALB/c mice using three multiple-epitope vaccines [27]. Additionally, the lack of appropriate cytokine stimulation and the failure to stimulate the coveted innate immune feedback further contribute to the suboptimal performances of these vaccines. A truly effective vaccine must have the ability to stimulate specific immune responses against HIV-1 by enhancing both CTL and HTL activities, as CTL-mediated feedback expresses a key role in the management of viral infections. Furthermore, the significance of HTL-mediated immunity cannot be ignored since it is vital for supporting antibody immune responses and encouraging a functional CD8+ cytotoxic T lymphocyte (CTL) response, which, in turn, provides protection against the virus and a decrease in viral load [28]. It is quite possible to improve the design of immunogens for HIV-1 vaccines by developing innovative immunoinformatics methods for the study of HIV-1 and the discovery of multi-functional T-cell epitopes, mainly when paired with in vivo studies [29]. Strong cellular and HTL feedback can be elicited using multiple-epitope-based vaccines. Notably, the methods used in these studies have paved the way for the suggestion of a multiple-epitope vaccine that can successfully provoke broadly neutralizing antibodies, stimulate the production of the appropriate cytokine like IFN-γ, and trigger the desired innate response via docking with TLRs, complemented by the addition of an adjuvant Gb-1 at the vaccine’s C-terminal. Due to the encouraging results of several studies, in silico techniques have become more important in the construction of multiple-epitope vaccines. However, a multiple-epitope vaccine against the Onchocerciasis disease caused by microfilaria was effectively developed in an in silico investigation [30]. Similar to this, a multiple-epitope vaccination against MERS was created using an immunoinformatic technique [31]. A multiple-epitope vaccine against brucellosis showed encouraging T-cell responses [32]. Furthermore, pre-clinical studies using animal models and a multiple-epitope Epstein–Barr virus vaccination showed encouraging outcomes [33]. Similar to our current work, a multiple-epitope vaccine that is specially designed to target HIV-1 has also been created [34].

The current immunoinformatic strategy was used to design a vaccine against HIV-1, and the focus was directed toward the envelope glycoprotein, specifically gp120, which involves attachment to the host receptor CD4 and enhances the virulence. Glycoproteins, prominently displayed on the surfaces of viruses, have long been prime candidates for vaccine development, as these surface antigens serve as the initial point of contact with the host’s immune cells. The current trial embraced a non-traditional and efficient procedure rooted in computational biology, harnessing the wealth of genomic data available, to design a multiple-epitope-based vaccine targeting diverse strains of HIV-1. Given the proven efficacy of immunoinformatic techniques in vaccine design, the principal goal of this work was to design a vaccine that might lessen the worldwide burden of various cancers brought on by the virus. The 3D structure and predicted epitopes were built using the consensus sequence. Several possibilities were produced when lineal epitopes for MHC-I and -II binding areas were predicted, and ElliProt was used for non-linear prediction. Furthermore, the 3D visualization of gp120 highlighted epitopes that may be recognized by antibodies. Considering that lower IC50 values imply better affinity, the use of a QSAR model, which forecasts IC50 values, made it easier to categorize peptides according to their affinity for the major histocompatibility complex (MHC).

T cells, which include both HTLs and CTLs, are essential for triggering an efficient immune response and protecting the host against viral infections. However, vaccines targeting CTL responses alone have shown lesser effectiveness compared to those targeting both CTLs and HTLs. Additionally, because of their specific qualities, these multiple-epitope vaccines have significant benefits over conventional and single-epitope vaccinations: (i) Different MHC class T-cell TCRs are capable of recognizing a wide variety of self and MHC class II epitopes that come from different T cell subsets. (ii) Interacting humoral and cellular immune responses can be triggered concurrently by overlapping CTL, HTL, and B-cell epitopes, promoting an all-encompassing and well-coordinated defense. (iii) Including an adjuvant in the formulation of the vaccine enables persistent immune feedback with increased immunogenicity. (iv) The difficulties resulting from in vitro antigen expression and pathogen culture can be avoided by eliminating the accompanying difficulties. Using the IEDB prediction service, T-cell epitopes that can recognize a variety of MHC class I and class II molecules were carefully chosen to further boost the immune feedback. In Tond epitopes with a greater affinity for binding and low percentile rankings (IC50), this selection method looked for them. On the other hand, exposed antigenic epitopes immediately recognize B-cell receptors (BCRs), which then stimulate the production of antibodies that are specific to those epitopes. This study used the IEDB prediction service to find linear or continuous B-cell epitopes. Overall, 106 T-cell and B-cell epitopes were ultimately chosen after the first screening phase based on the favorable results. The most promising choices among these epitopes, which had highly antigenic qualities while being free of allergens, toxins, and homologies with the human proteome, were subsequently chosen after going through multiple rigorous rounds of screening. Additionally, the HTL epitopes’ capacities to elicit cytokine responses, including IFN-γ, IL-4, and IL-10 responses, were evaluated. Gb-1, an adjuvant, was used to boost the vaccine’s strong immunogenicity. Using the linkers GTG, GSG, GGGGS, and GGTGG, the epitopes were joined at the proper locations. Adjuvants and linkers were used to strengthen the vaccine’s structure and improve its immunogenicity, antigenicity, and durability.

Long peptides have demonstrated superior efficacy in eliciting immune responses compared to short peptides [35]. However, there remains uncertainty regarding whether artificially anticipated multiple-epitope constructs, comprising T- and B-cell epitopes, can be adequately displayed and processed to activate targeted immunity. Consequently, an essential aspect in enhancing multiple-epitope-based vaccines is the ability to predict TAP transport and proteasomal cleavage. According to the findings from the NetCTL 1.2 server, it appears that all chosen epitopes, including CD8+ and CD4+ T-cells, are accessible for immune feedback during the antigen processing and presentation carried out by competent APCs. Evaluating the immune feedback triggered by these epitopes is crucial for their judicious preference in vaccine design. Since specific cytokines can be induced by particular residues and motifs within an epitope, employing in silico cytokine prediction tools provides a comprehensive overview of the capacity of T-cell epitopes to stimulate heterogenous cytokines in a straightforward, rapid, and cost-effective way compared to in vitro and in vivo immunological evaluation [36]. IFN-γ is a cytokine that serves as the hallmark of adaptive and innate immunity, displaying antiviral, immune regulatory, and anti-tumor properties. IFN-γ secretion plays a pivotal role in the Th1 response and is crucial for diminishing the viral load of HIV-1 [37]. In this particular investigation, we evaluated the capacity of IFN-γ and the production of cytokines for each selected HTL epitope. To accomplish this, we employed the IFNepitope server to forecast peptides that could induce IFN-γ through binding to MHC class II molecules. The majority of our HTL epitopes demonstrated positive induction of IFN-γ and cytokine production, as indicated by their predicted SVM scores. The production of IFN-γ is closely related to the immunogenicity of HIV-specific T-cells and the elicitation of Th1 responses [38]. Some studies described that IL-10 possesses anti-HIV activity by inhibiting the release of inflammatory cytokines [39]. Furthermore, additional research has shown that T-cells that secrete IL-10 contribute to the reduction in HIV replication in pregnant women [40].

Vaccine immunogens must be produced to combat the antigenic diversity of HIV-1 and the various HLA tissue types. To increase the population coverage rate in our investigation, we used peptides with different epitopes and numerous HLA binding specificities. MHC class I epitope population coverage was 90.23%, whereas MHC class II population coverage was 72.95%. Moreover, particularly in areas with a high prevalence of HIV-1, the multiepitope structures showed a significant cumulative population coverage. The size of the final designed epitopes showed an epitope conservation score of 75% among the M group of HIV-1 subtypes. The probability of viral immune evasion was decreased by high conservation across HIV-1 subtypes, which also offered wider protection. Utilizing the Vaxigen, AllerTOP v.2.0, and Toxinpred servers, respectively, the antigenicity, allergenicity, and toxicity of the predicted epitopes and the final construct of the HIV-1 gp120 vaccine sequence were evaluated. The outcomes demonstrated the antigenicity, non-allergenicity, and non-toxicity of the vaccine protein. The ProtParam program from the ExPASy website was used to examine the physicochemical characteristics of each predicted epitope and the proposed construct. With a high pI value of 10.13, the HIV-1 gp120 vaccine demonstrated acceptable stability and is considered to exist within the normal range. The vaccine showed a half-life beyond the 10 h in a prokaryotic *E. coli* culture system and 30 h in mammalian reticulocytes in vitro, indicating the possibility of the stable and scalable production of the vaccine in this system. A GRAVY value of -0.741 further demonstrated the vaccine’s hydrophilic nature and increased water solubility. High solubility was predicted via SolPro for the HIV-1 gp120 vaccine.

HIV-1 gp120 constructs were 3D-modeled using the RoseTTAFold web tool and then refined using the GalaxyRefine tool to make sure the protein closely matched its native or natural structure. Our results showed that after refining the vaccine construct’s, the final accuracy and the anticipated 3D structures’ quality both improved. The Ramachandran plot and the Z-score observed during the 3D and refined structure assessment of the HIV-1 gp120 vaccine validated the good outcomes. This analysis indicated that the vaccine possessed a structurally adequate form and should be effective as a vaccine. The vaccine’s Z-score was -6.02, which is in the acceptable range for experimentally validated X-ray crystal structures of proteins. Notably, the highest concentration of amino acids was observed in the analysis’ preferred regions [41]. Additionally, protein–protein docking analysis between TLRs and our predicted constructs was carried out. By identifying pathogens and subsequently eliciting adaptive immune feedback, TLRs have a significant impact on activating the innate immune system [42]. TLR2 and TLR4 detect virus structural proteins and release inflammatory cytokines as a result. Additionally, TLR3 is in charge of the activation of dendritic cells caused by HIV-1. [43].

In our study, an in silico assay was conducted to examine the correlation between the HIV-1 gp120 construct and various TLRs, as mentioned in the methodology. The findings showed low energy scores and significant binding affinity between the vaccine design and TLRs. Contact investigation using contact maps displayed different patterns of interchain residue-to-residue association in the TLR-vaccine complexes. Specifically, the TLR2-vaccine complex exhibited a greater number of interchain contacts in various domains of the proteins. Three particular areas on the contact map in the TLR3 and TLR8 vaccine complexes showed interchain interactions between various domains. Fewer connections were seen in the TLR4–vaccine complex, indicating a less robust link between the TLR and vaccine chains. Interchain interactions in the TLR5–vaccine complex were restricted to closely related residues, but the TLR8–vaccine complex showed different vaccine chain residues making contact with the TLR chain. These findings suggest that the designed vaccine constructs have the potential to activate TLRs and downstream pathways, leading to the production of pro-inflammatory cytokines to counter the HIV-1 infection. A potent HIV-1 therapeutic vaccine should stimulate cellular and humoral immune feedback. A TRIF-dependent signaling cascade that is started by TLR-3 activation can lead to the transcription of inflammatory genes. [44]. This stimulation in DCs leads to their maturation into potent immunostimulatory cells capable of productively cross-priming T-cells [43]. In the context of HIV-1 infection, the stimulation of DCs is reliant on TLR-3 activation [45]. On the other hand, TLR-4 activation can result in the induction of IL-6, which has the potential to re-emerge the virus from its dormant stage [46]. Additionally, TLR-10 has been associated with enhanced HIV-1 infection [47].

MD simulations were conducted to ensure the binding efficiency and equilibrium of the vaccine–receptor complex. These simulations allowed us to observe the correlation of the vaccine in association with the TLR receptors over time. The results indicated that the vaccine design was able to effectively occupy the TLR receptors with minimal energy, suggesting a strong binding affinity. Additionally, normal-mode evaluation performed using iMODS showed that the vaccine complex exhibited elevated eigenvalues, indicating a lower extent of deformability. This suggests that the vaccine construct is relatively rigid and less prone to conformational changes. The deformability graph further supported this observation, indicating that the vaccine construct is likely to maintain its structural integrity and stability. Furthermore, atomistic simulations provided valuable insights into the stability and conformational dissimilarities of the physiological systems. By examining several descriptors derived from the trajectory data of molecular dynamics simulations, we were able to study the conformational variations in and stability of the vaccine–receptor complex in a more precise manner [48]. These simulations provide a deeper understanding of the dynamic manner and structural characteristics of the vaccine construct in complex with the TLR receptors.

To enhance the mRNA of the vaccine, the JCAT was employed, with the *E. coli* strain K-12 selected as the cell culture system. This tool was used to analyze the translation capacity of the HIV-1 gp120 vaccine. The results yielded a CAI value of 0.60 and a GC content of 61.90%. The current values were deemed acceptable, as a CAI value above 0.60 and a GC content ranging from 30 to 70% are considered favorable scores. During codon optimization in the pET plasmid, *NcoI* and *XhoI* restriction enzymes were utilized to cleave the N and C termini, respectively. The presence of 6xHis tags in the cloned plasmid allows for the post-translational purification of the vaccine. Furthermore, the RNAfold (version 2.1.0) software was employed to analyze the secondary structure of the HIV-1 gp120 mRNA. The software produced a minimum free energy value of -378.55 kcal/mol, indicating a more stable vaccine within the body. This stability is significant during vaccine construction. Moreover, in silico assessments of the host immune responses to our designed multiple-epitope vaccine demonstrated significant findings. The C-ImmSim tool predicted the activation of B cells and T cells, leading to prolonged memory. The presence of IgG1 and IgG2 showed Th1 and Th2 responses to HIV-1 antigens, suggesting significant protection against HIV-1 infection. However, results from the ICM server indicated a significant increase in the level of Th1 cells correlates with an elevation in the level of CTL. The current findings show that the vaccine construct, encompassing the immunogenic B- and T-cell epitopes of gp120, holds promise for the development of multiple-epitope vaccines against HIV-1. However, further in vitro and in vivo investigations are compulsory to fully assess the potential of our designed multiple-epitope-based vaccines for combating HIV-1.

## 4. Methodology

### 4.1. Protein Sequence and Multiple-Sequence Exploration

GenBank was initially searched for gp120 protein sequences connected to HIV. To determine the mutant and conserved portions of the gp120 protein, these sequences were then submitted to a multiple-sequence alignment using the STRAP program. The muscle server was used to create a consensus sequence.

### 4.2. B-Cell Epitope Prediction

B-cell epitopes are key players in vaccine design as they are recognized by the immune system. The current research employed two tools, namely IEDB Bepipred linear epitope prediction tool and BepiPred-2.0, to identify linear B cell epitopes within the gp120 protein sequence. Default thresholds were utilized during the analysis [49,50]. BepiPred-2.0 is a tool that anticipates B cell epitopes by employing a random forest algorithm with epitope trains obtained from antibody–antigen protein structures. This procedure combines a sizable number of linear epitopes acquired from the IEDB network with sequence-based epitope prediction exploiting 3D structures. In addition, the B-cell epitopes discovered by IEDB and BepiPred-2.0 were validated using iBCE-EL. We used a web-based analysis tool for linear B-cell epitopes called iBCE-EL. It utilizes a mix of dipeptide and physicochemical characteristics, very randomized tree and gradient boosting techniques, and a combination of amino acid configuration and physicochemical properties as input features. iBCE-EL forecasts a peptide’s class and probability values when given the peptide [51]. It is important to note that almost 90% of B-cell epitopes are thought to be discontinuous, which means they are composed of sequences that are close while being far from one another in terms of long-distance pathogenic protein sequences. In the final revised vaccination 3D structural model, we used the ElliPro tool to analyze the conformational B-cell epitopes. The geometrical characteristics of protein structure were analyzed via ElliPro. ElliPro has the greatest AUC value of 0.732 among conformational B-cell epitope prediction methods, making it a very accurate technique for detecting antibody epitopes in protein antigens.

### 4.3. Cytotoxic T-Cell Epitope

To identify MHC-I-binding alleles, both commonly occurring and less frequent ones, the epitopes were analyzed using the NN-align tool in the IEDB analysis tool. The identification of MHC-I-binding alleles was based on the following parameters such as a peptide length of 8–12 amino acids and an IC50 value of less than 200. Peptides with IC50 values below 50 nM were regarded as occupying stronger affinity, while those below 500 nM showed moderate affinity, and those below 5000 nM had a poor affinity in this context. A lower IC50 value, therefore, denotes a greater affinity. The IEDB server is a comprehensive resource that was also utilized to predict the processing score, proteasomal cleavage, TAP value, and MHC-I-binding value of the selected epitopes and their corresponding alleles via the NN-align algorithm.

### 4.4. Class I Immunogenicity Assessment

MHC-peptides with potential for immunogenicity within the infected host cell were evaluated by using the IEDB MHC I immunogenicity prediction server. The server’s default settings were used for the evaluation of the shortlisted epitopes. The epitopes with a high immunogenicity value were then chosen for additional experiments.

### 4.5. Helper T Cell Epitope

Helper T-lymphocytes (HTLs), which identify foreign antigens and stimulate B-cells and cytotoxic T-cells, are essential for the immune system’s response to infectious diseases. The Immune Epitope Database was employed to analyze MHC class II T-cell epitopes. Based on either the percentile rank or the MHC binding affinity, the server predicted the binders. We used the IEDB-recommended combinatorial method to determine HTL epitopes. This strategy incorporated NN-align, SMM-align, CombLib, Sturniolo, and NetMHCIIpan techniques [52]. The final selection of epitopes was based on the epitopes’ scores (lower scores indicating better binding), capacity to release IFN-γ, ability to produce emergent characteristics, and an IC50 value of less than 500 nm.

### 4.6. Selection of Top Epitopes

The selection of thresholds for the epitope selection process was based on previous studies. The aim was to strike a balance between achieving a high sensitivity to obtain a large number of high-quality epitopes and maintaining specificity. Individual filters were applied to each epitope based on the results of various tests conducted using a dataset of experimentally confirmed epitopes. This dataset primarily consisted of HIV-1 data, as it provided a comprehensive collection of immunogenic and non-immunogenic epitopes derived from a meta-analysis of multiple studies focusing on MHC class I epitopes.

### 4.7. Proteasomal Cleavage/TAP Transport

NetCTL 1.2 was employed for proteasomal cleavage and transporter-associated antigen processing (TAP). The website uses an algorithm that provides predictions for the efficiency of TAP transport, MHC class I binding, and proteasomal C-terminal cleavage. The default parameters gave TAP transport ability, with a weight of 0.05, and C-terminal cleavage, with a weight of 0.15.

### 4.8. IFN-γ Cytokine Inducer Prediction

The website server is used to predict the epitopes that cause IFN-γ cytokine production. The intracellular Th1 immune responses that IFN-γ stimulates are known to produce antiviral action in both the innate and adaptive immune systems. This service was used to anticipate MHC class II-binding peptides that may cause CD4+ T cells to produce IFN-γ. To prioritize IFN-γ over other cytokines, the server settings were changed to choose the Support Vector Machine (SVM)-based approach.

### 4.9. Determination of Population Coverage

The coverage rate of particular epitopes in the population was measured using the population coverage tool on the IEDB website. The global human population was evaluated via the given tool. Predicting the right epitopes for various HLA bindings can be aided by estimating population coverage. Varied ethnic groups may have varied frequencies of epitopes with stronger HLA binding affinities, which aids us in overcoming the drawbacks of MHC restriction in T-cell responses. In this study, the MHC-I and MHC-II HLA binding alleles were assessed for the gp120 protein. Moreover, the IEDB online server epitope conservation analysis tool was employed to anticipate the conserved cross-reactive epitopes by determining the identity of a specific peptide sequence among various HIV-1 subtypes in group M.

### 4.10. Antigenicity, Allergenicity, and Solubility Analysis

The AntigenPro server and the VaxiJen 2.0 server were employed to confirm antigenicity for the final vaccine and its components. To convert protein sequences into constant vectors of primary amino acid characteristics, VaxiJen uses the auto cross-covariance (ACC) transformation [53]. AntigenPro, on the other hand, is a pathogen-independent, sequence-based predictor of protein antigenicity [54]. The AllergenFP 1.0 and the AllerTOP 2.0 tools were employed to analyze the allergenicity of the final vaccine and its components. To differentiate allergens from non-allergens, AllergenFP employs a binary classifier. Five E-descriptors characterize the dataset, and the strings are converted into constant vectors using the ACC transformation [55]. The ACC transformation and E-descriptors are also used by AllerTOP. The SolPro server and the Protein-Sol server were used to assess solubility. SolPro uses SVM to predict a protein sequence’s solubility with a tenfold cross-validation accuracy of more than 74%. Protein-Sol, on the other hand, is based on data from a cell-free production system for Escherichia coli protein solubility [56].

### 4.11. Toxicity and Physicochemical Properties Assessment

The ToxinPred tool was employed to anticipate the toxicity of the final vaccine and its components. ToxinPred ranks toxicity and non-toxicity using an SVM model [57]. The ExPASy ProtParam tool was employed to forecast the physicochemical properties of the final vaccine and its components. Hydropathicity, charge, half-life, instability index, pI (theoretical isoelectric point value), and molecular weight are among these qualities [58].

### 4.12. Hydropathy Analysis of Epitopes

In the case of HIV-1 vaccines, all epitopes must possess a hydrophilic nature, meaning they should be present on the surface. This is because hydrophilic epitopes can provoke immune feedback within the host cell. To analyze the hydrophobicity of the epitopes, the GRAVY (grand average of hydropathy) score was measured through the ProtParam server. Calculating the total hydropathy values of all the amino acids in the protein and dividing that number by the total number of amino acid residues yields the GRAVY score. A positive GRAVY score specified a hydrophobic protein, while a negative value specified the presence of a hydrophilic region.

### 4.13. MHC Restricted Alleles through Cluster Analysis

We used the IEDB server to narrow down the selection of epitopes restricted to MHC class I and class II. To validate the predictions, we also employed the MHCcluster v2.0 server, which provided additional verification. This server presents the relationship between peptides and HLA functionality through a static heat map visualization [59].

### 4.14. Multiepitope Subunit Vaccine Design

The epitopes that displayed the most promising characteristics, including high antigenicity, non-allergenic and non-toxic characteristics, a lack of homology to the human proteome, and conservation across selected strains, were carefully examined. Their potential to stimulate cytokines was assessed, and only the HTL epitopes competent in eliciting cytokine were chosen for vaccine development. The most promising epitopes that fulfilled the required standard and passed the above analysis were considered for constructing a multiple-epitope vaccine against HIV-1. To design the vaccine, the best-selected B-cell, CTL, and HTL epitopes were connected using an adjuvant Gb-1 (GP-2 binding peptide) sequence and different linkers. The Gb-1 peptide was identified in our laboratory through the utilization of the phage display screening method. It was discovered that the Gb-1 peptide possesses immune stimulatory properties and specifically triggers a Th2 immune response. The adjuvant plays a crucial role in enhancing the antigenicity, immunogenicity, stability, and longevity of the vaccine. The adjuvant was potentially linked to the epitopes using GTG linker and the inclusion of the Gb-1 sequence improves the ability of the immune feedback induced by the vaccine. Subsequently, the B-cell, CTL, and HTL epitopes were linked in a consecutive sequence using GTG, GSG, GGTGG, and GGGGS linkers.

### 4.15. Assessment of Physicochemical Properties of the Vaccine Construct

A crucial aspect of vaccine development is ensuring higher antigenicity, which guarantees the identification of the vaccine by the host immune system, leading to the stimulation of immune cells and succeeding immune response [60]. To make sure that the vaccination does not cause an allergic reaction in the host, allergenicity testing must be carried out. Additionally, to guarantee the safety and potency of the vaccine, a thorough assessment of several physicochemical variables is required. VaxiJen v2.0, with an accuracy criterion of 0.4, was employed to observe the antigenicity of the created vaccine. Using AlgPred and AllerTop v2.0, the allergenicity of designed vaccine was also evaluated. The AlgPred service uses a variety of techniques to assess antigenicity and forecast probable allergenicity by comparing the resemblance of common epitopes in protein regions.

The allergenicity of designed vaccine was predicted using the MEME/MAST motif prediction in AllerTop v2.0. ProtParam was once more used to ascertain the physicochemical characteristics of the vaccines, including variables like the pI value, half-life, and the GRAVY value. Additionally, the SOLpro tool of the SCRATCH protein predictor was used to forecast the solubility of the vaccine construct while maintaining the default settings throughout the prediction, and the results were confirmed using the Protein-Sol tool. The solubility factor was essential in making sure that the vaccine was sufficiently soluble after being given to the host. A vaccine’s efficacy might be harmed even if it is very effective if it clumps into insoluble substances. Protein-Sol uses a quick sequence-based procedure to determine the findings, whereas the SolPro service uses the SVM method to predict solubility. As a result, it is possible to estimate the solubility of protein sequences using any of these services with confidence [56].

### 4.16. Secondary and Tertiary Structure Analysis of the Vaccine

As a protein’s functioning depends on a certain structural conformation, it is essential to assess the secondary and tertiary structures of a designed vaccine. Using the online PRISPRED program, the secondary structure of the vaccine was ascertained after antigenicity and allergenicity testing while maintaining all the default settings. PRISPRED is a server designed to assess the secondary structure of biomolecules, as well as their transmembrane topology, helices, folds, and domain recognition [61]. Two-dimensional (2D) structural analysis was carried out by utilizing the SOPMA and Phyre2 servers for correlation and extra confirmation to further validate the results. To construct the 3D design of the vaccine, we used the RoseTTAFold (BOINC version 7.6.22) software. RoseTTAFold is a neural network-based method that incorporates a “three-track” approach. It considers patterns in protein sequences, interactions between amino acids within the protein, and potential 3D structures. This comprehensive approach allows the complex to inclusively link the chemical components of a protein and its folded structure. RoseTTAFold has achieved high accuracies comparable to those of DeepMind. In the RoseTTAFold architecture, information flows back and forth between the one-, two-, and three-dimensional levels, enabling a holistic understanding of a protein’s structure [62].

### 4.17. Refinement and Validation of 3D Vaccine Structure

The computation of vaccine design using computer-based methods is important, particularly when there is limited experimental evidence available. While designing 3D models is not always sufficient for ensuring accuracy in biomedical applications, it is important to have experimental data to verify the accuracy. Through the process of 3D structure refinement, it became feasible to enhance the fidelity of primitively designed structures and rectify local flaws while preserving the vaccine’s fundamental structure as closely as possible. The GalaxyRefine tool was used to improve the 3D vaccination architecture. This server employs a CASP10-tested approach for refinement and dynamics simulation, which resulted in upgraded structures [63]. Moreover, achieving precise and uniform refinement of 3D vaccine models remains a difficult task, especially when working with high-resolution data. Therefore, to validate the accuracy of the vaccine design, Ramachandran plots were designed using the PROCHECK server [64]. These plots analyzed the allowed and disallowed dihedral angles (psi and phi) of the amino acid configurations, taking into account the van der Waals radius of the side chain. Additionally, the ProSA-web tool was utilized, which employs various statistical methods to produce a Z-score that serves as a measure of protein structure validation. The Z-score indicates the standard of the protein structure being analyzed. By comparing the Z-score of the query protein with the range of Z-scores found in the PDB database for experimentally determined protein chains, we could determine the quality and consistency of the query protein’s structure [65].

### 4.18. Evaluation of Vaccine Disulfide Engineering

A key component of vaccine design is using multiple molecular interactions to increase the stability of protein vaccines. Disulfide by Design 2 v12.2 was employed to analyze the disulfide design of vaccine protein. This server’s purpose is to identify probable locations in a protein structure where disulfide linkages are more likely to occur. To predict protein structure, the tool uses computational methods [66]. Its technique uses a geometric framework built from natural disulfide links to precisely evaluate the x3 torsion angle based on the 5th Cbeta–Cbeta distance.

### 4.19. Vaccine Construct Docking with TLRs

Docking serves as a structural configuration for both drug design and basic investigations into protein interplay. It involves predicting the structure of a complex by combining the individual protein structures through protein–protein docking. In the current experiment, protein–protein docking was examined by docking it with different TLRs, including TLR-2, 3, 4, 5, 8, and 9 (PDB ID: 2Z7X, 1ZIW 3FXI, 3J0A, 3W3G, and 3WPB). The 3D structures of both TLRs and the HIV-1 gp120 construct were then refined for energy using PyMOL v2.3.4 software. To enhance the accuracy of predictions, various online docking tools were employed for protein–protein docking assessment. The PDB structure of the vaccine, TLRs, and HIV-1 gp120 experienced the removal of all water molecules. During the docking analysis, the 3D structure was observed using the Chimera V 1.13.1 server. Next, the HIV-1 gp120 constructs, along with TLR2 and TLR8, were submitted to the HADDOCK server to evaluate their interaction areas. The complexes with the highest rankings were selected based on the lowest intermolecular binding and the lowest mean RMSD from the HADDOCK analysis, representing the whole-molecular HIV-1 gp120–TLR interaction.

The clusters of docked complexes were arranged using ClusPro 2.0 in the second phase of docking, occupying the center and lowest energy ratings. Here, E = 0.40Erep + 20.40Eatt + 600Eelec + 1.00EDARS, and this equation was used with the ClusPro tool to calculate the energy score [67]. A higher binding affinity was indicated by a lower energy score. Molecular mechanics/generalized Born surface area (MM-GBSA) research was carried out via docking, using all default settings. Following a successful docking procedure, visualization and MD simulation investigations were used to further evaluate the TLRs docked with the vaccine construct. The Discovery Studio Visualizer was used to visualize the docked complexes. PDBsum was used to conduct a thorough investigation of protein–protein interplay inside the complexes [68]. H-band formation was evaluated using the LigPlot+ program.

### 4.20. Molecular Dynamics Simulation of the Vaccine-Receptor Complex

iMOD was employed to run the molecular dynamics simulation. Such modes may be explored using iMODS, which also creates workable transition routes between two homologous structures [69]. By using normal mode analysis (NMA), the server computes internal attributes to evaluate the stability of proteins. The main-chain deformability plot, B-factor values, eigenvalue, covariance matrix, and elastic network model are some of the characteristics used to represent protein stability. Understanding the protein’s stability and structural dynamics is possible through such analysis.

### 4.21. Codon Optimization and In Silico Cloning of the Vaccine Construct

Reverse translation of the desired protein was carried out to find a suitable DNA sequence that might transcribe our peptide vaccine. The intended organism was then given the altered DNA sequence after codon optimization, which allowed it to successfully express the target protein utilizing the codons from the altered DNA sequence. The Java Codon Adaptation Tool (JCat) server was employed to carry out the codon adaptation procedure for the proposed vaccination protein [70]. The prokaryotic *E. coli* strain K-12 was preferred as the target organism. During the codon adaptation process, certain considerations were made to bypass rho-independent transcription terminators, prokaryotic ribosome-binding sites, and cleavage sites for the restriction enzymes *NcoI* and *XhoI*. Additionally, the N- and C-termini of the optimized DNA sequence were positioned at the combined *NcoI* and *XhoI* restriction sites. Using the SnapGene restriction cloning tool, the modified DNA sequence was implanted between the *NcoI* and *XhoI* restriction sites of the pET-28a (+) vector [71]. The DNA sequence of the pET-28a (+) vector included a single 6×His tag. The recombinant protein’s solubilization and efficient affinitative purification were made easier by the presence of this tag [70].

### 4.22. Immune Simulations of Vaccine Construct

To model and forecast the immune feedback to the recombinant HIV-1 gp120 construct, the C-ImmSim server was used. It used a position-specific scoring matrix (PSSM) to evaluate immunological interactions through an agent-based model and machine learning approaches. The simulation steps domain was adjusted to 1050 to reflect the 4-week gap between the first and second doses of the vaccination. Each time step, which equaled eight hours in actual time, was set at 1 and 84 and applied to two injections. Other settings remained at their preset values during the study.

## 5. Conclusions

Bioinformatics plays a key role in predicting immunogenic epitopes, which are crucial for designing effective vaccines. The current study developed a multiple-epitope vaccine aimed at targeting HIV-1 gp120 structural proteins. The vaccine was designed by combining potential linear B cell epitopes, cytotoxic T lymphocyte epitopes, and helper T lymphocyte epitopes. Initially, we conducted screening for the antigenicity, toxicity, and allergenicity of the selected epitopes. Subsequently, we utilized the adjuvant Gb-1 and various linkers to attach these epitopes. The physicochemical profile and validation of the 3D structure of our vaccine demonstrated positive results across the selected parameters. Molecular docking studies revealed that the final construct exhibited strong binding affinity with receptors TLR3, TLR4, TLR5, TLR8, and TLR9. Furthermore, analyses including immune simulation and molecular dynamics simulation provided satisfactory results. However, further investigation is required to ascertain the effectiveness of anti-HIV-1 immune responses, including antibodies and cytokines against the polypeptide, in reducing HIV-1 infection in animal models.

## Figures and Tables

**Table 1:** Different immunoinformatic servers were employed in the development of the vaccine.

Parameter	Server Name	Server Link
Consensus sequence	muscle server	https://www.ebi.ac.uk/Tools/msa/muscle/ (accessed on 11 December 2023)
B-cell prediction	IEDB	http://tools.iedb.org/bcell/ (accessed on 11 December 2023)
B-cell prediction	BepiPred-2.0	http://www.cbs.dtu.dk/services/BepiPred/ (accessed on 11 December 2023)
B-cell prediction	iBCE-EL	http://thegleelab.org/iBCE-EL/ (accessed on 11 December 2023)
B-cell prediction	ElliPro	http://tools.iedb.org/ellipro (accessed on 11 December 2023)
CTL prediction	IEDB	http://tools.iedb.org/mhci/ (accessed on 11 December 2023)
Immunogenicity	IEDB	http://tools.immuneepitope.org/immunogenicity/ (accessed on 12 December 2023)
HTL prediction	IEDB	http://tools.iedb.org/mhcii/ (accessed on 11 December 2023)
TAP and proteasome	NetCTL 1.2	https://services.healthtech.dtu.dk/services/NetCTL-1.2/ (accessed on 12 December 2023)
IFN-γ prediction	IFNEpitope	http://crdd.osdd.net/raghava/ifnepitope/index.php (accessed on 12 December 2023)
Population coverage	IEDB	http://tools.iedb.org/population (accessed on 12 December 2023)
Antigenicity	VaxiJen 2.0	http://www.ddg-pharmfac.net/vaxijen/VaxiJen/VaxiJen.html (accessed on 12 December 2023)
Antigenicity	AntigenPro	http://scratch.proteomics.ics.uci.edu(accessed on 12 December 2023)
Allergenicity	AllergenFP 1.0	http://ddg-pharmfac.net/AllergenFP/ (accessed on 12 December 2023)
Allergenicity	AllerTOP 2.0	https://www.ddg-pharmfac.net/AllerTOP/ (accessed on 12 December 2023)
Solubility	SolPro	http://scratch.proteomics.ics.uci.edu (accessed on 12 December 2023)
Solubility	Protein-Sol	https://protein-sol.manchester.ac.uk (accessed on 12 December 2023)
Toxicity	ToxinPred	http://crdd.osdd.net/raghava/toxinpred/ (accessed on 12 December 2023)
Physicochemical properties	ExPASy ProtParam	https://web.expasy.org/protparam/ (accessed on 12 December 2023)
MHC cluster analysis	MHCcluster v2.0	https://services.healthtech.dtu.dk/services/MHCcluster-2.0/ (accessed on 13 December 2023)
Secondary structure	PRISPRED	http://bioinf.cs.ucl.ac.uk/psipred/ (accessed on 13 December 2023)
Secondary structure	SPOMA	https://prabi.ibcp.fr/htm/site/web/app.php/home (accessed on 13 December 2023)
Secondary structure	Phyre2	http://www.sbg.bio.ic.ac.uk/~phyre2/html/page.cgi?id=index (accessed on 13 December 2023)
3D structure	RoseTTAFold	https://robetta.bakerlab.org/ (accessed on 13 December 2023)
Structure refinement	GalaxyWEB	http://galaxy.seoklab.org/ (accessed on 14 December 2023)
Ramachandran plot	PROCHECK	https://saves.mbi.ucla.edu/ (accessed on 14 December 2023)
Z-score	ProSA-web	https://prosa.services.came.sbg.ac.at/prosa.php (accessed on 14 December 2023)
Disulfide engineering	Design 2 v12.2	http://cptweb.cpt.wayne.edu/DbD2/ (accessed on 14 December 2023)
Protein prediction	Chimera V 1.13.1	https://www.cgl.ucsf.edu/chimera/olddownload.html (accessed on 14 December 2023)
Protein docking	HADDOCK	https://wenmr.science.uu.nl (accessed on 14 December 2023)
Protein docking	ClusPro 2.0	https://cluspro.bu.edu/login.php (accessed on 14 December 2023)
Protein–protein interaction	PDBsum	https://www.ebi.ac.uk/thornton-srv/databases/pdbsum/ (accessed on 14 December 2023)
Protein interaction	LigPlot+	https://www.ebi.ac.uk/thornton-srv/software/LigPlus/ (accessed on 15 December 2023)
Molecular dynamic simulation	iMOD	http://imods.chaconlab.org (accessed on 15 December 2023)
Codon optimization	JCat	http://www.jcat.de/ (accessed on 15 December 2023)
Immune simulation	C-ImmSim	https://kraken.iac.rm.cnr.it/C-IMMSIM/ (accessed on 15 December 2023)
Cloning	SnapGene	https://www.snapgene.com/ (accessed on 15 December 2023)

**Table 2:** Linear gp120 predicted B cell epitopes.

Length	Peptide	Antigenicity	Allergenicity	Toxicity	GRAVY
59–72	*VNVTENFNMWKNDM*	−0.1226	Yes	No	−0.821
105–125	DLKNDTNTNSSSGRMIMEKGE	0.0921	No	No	−1.395
137–148	*IRGKVQKEYAFF*	0.5359	No	No	−0.408
173–182	ITQACPKVSF	0.9975	Yes	No	0.53
272–282	NNNTRKRIRIQ	0.1835	No	No	−2.1
334–343	*KQSSGGDPEI*	0.597	No	No	−1.39
404–410	*KAMYAPP*	0.6789	No	No	−0.414
461–480	*VKIEPLGVAPTKAKRRVVQR*	1.1063	No	No	−0.39
136–148	SIRGKVQKEYAFF	0.6778	No	No	−0.438
431–458	*GNSNNESEIFRPGGGDMRDNWRSELYKY*	−0.0127	No	No	−1.607
460–480	VVKIEPLGVAPTKAKRRVVQR	0.9768	No	No	−0.171

The B-cell epitopes were predicted using IEDB, BepiPred 2.0, and iBCE-EL. Among these epitopes, the ones selected for the final vaccine construct are highlighted in italic characters. The selection of these epitopes was based on their antigenic properties, non-allergenicity, non-toxicity, and negative GRAVY values. Additionally, all epitopes underwent assessment for surface accessibility and flexibility to ensure their suitability.

**Table 3:** CTL gp120 epitopes predicted using the IEDB database server.

Length	Peptide	Antigenecity	Allergicity	Toxicity	GRAVY	IC50	PCC	TAP
469–478	*APTKAKRRVV*	0.7196	No	No	−0.71	27	0.8464	0.1160
190–199	CAPAGFAILK	1.0451	Yes	No	1.31	20.23	0.9696	0.9490
129–138	CSFNISTSIR	0.5758	No	No	0.32	14.17	0.9577	0.6440
268–276	CTRPNNNTR	0.7904	No	No	−2.222	56.31	0.8586	1.4690
384–392	DTITLPCRI	0.7402	No	No	0.478	66.1	0.5292	0.2640
384–393	DTITLPCRIK	0.958	No	No	0.04	72.76	0.6108	0.2640
240–249	EEVVIRSVNF	1.1433	No	No	0.41	91.67	0.9347	2.3800
195–203	FAILKCNNK	0.4852	Yes	No	0.067	56.88	0.6267	0.4890
25–33	FCASDAKAY	0.8015	Yes	No	0.133	13.55	0.3359	2.8650
363–372	*FNSTWFNSTW*	0.4342	No	No	−0.62	51.28	0.9706	0.8210
289–298	FVTIGKIGNM	1.7083	No	No	0.9	17.52	0.7651	0.3090
124–133	GEIKNCSFNI	0.4196	Yes	No	−0.13	78.86	0.7427	1.5630
467–475	*GVAPTKAKR*	1.8579	No	No	−0.8	94.58	0.8463	1.4590
173–182	ITQACPKVSF	0.9975	Yes	No	0.53	17.32	0.7506	2.6150
143–151	KEYAFFYKL	0.6017	No	No	−0.3	9.57	0.9763	1.1110
93–101	KLTPLCVSL	2.8013	Yes	No	1.233	41.51	0.9780	1.0430
93–102	KLTPLCVSLK	2.9246	Yes	No	0.72	23.49	0.9787	1.0430
89–97	KPCVKLTPL	1.5232	No	No	0.289	52.2	0.9698	0.7640
140–149	*KVQKEYAFFY*	0.6481	No	No	−0.58	37.23	0.5240	2.7460
179–187	KVSFEPIPI	2.4609	No	No	0.511	44.26	0.2494	0.7740
119–127	MIMEKGEIK	0.6697	Yes	No	−0.267	67.11	0.5022	0.7030
406–415	MYAPPISGQI	0.4699	No	No	0.35	56.26	0.4059	0.2210
420–428	NITGLLLTR	1.0874	No	No	0.678	42.71	0.8257	1.7230
261–270	NTSVEINCTR	1.2735	Yes	No	−0.6	7.84	0.8257	1.7230
142–151	*QKEYAFFYKL*	0.7239	No	No	−0.62	40.32	0.9763	1.0640
307–315	*RAKWNNTLK*	0.4822	No	No	−1.7	7.25	0.9319	0.7570
245–254	RSVNFTDNAK	1.794	No	No	−1.16	35.17	0.8873	0.6940
130–138	SFNISTSIR	0.9960	Yes	No	0.078	55.66	0.6153	1.6090
215–233	STVQCTHGI	0.5294	Yes	No	0.211	7.66	0.7631	0.6420
215–234	STVQCTHGIR	0.9619	Yes	No	−0.26	97.26	0.4286	1.6240
246–254	*SVNFTDNAK*	1.6628	No	No	−0.789	41.33	0.8873	0.5600
291–299	*TIGKIGNMR*	0.7225	No	No	−0.278	80.17	0.5029	1.4270
23–31	TLFCASDAK	1.1092	Yes	No	0.422	44.92	0.7740	0.5730
387–395	TLPCRIKQI	1.1073	No	No	0.122	72.38	0.8880	0.5160
174–182	TQACPKVSF	1.1348	Yes	No	0.089	25.69	0.7506	2.6040
262–270	TSVEINCTR	1.5595	Yes	No	−0.278	6.94	0.8257	1.5800
22–31	TTLFCASDAK	0.8339	Yes	No	0.31	18.26	0.7740	0.5850
216–224	TVQCTHGIR	1.2166	Yes	No	−0.2	51.81	0.4286	1.5680
141–149	VQKEYAFFY	0.9676	Yes	No	−0.211	42.18	0.9273	3.0580
141–150	VQKEYAFFYK	0.7979	Yes	No	−0.58	34.86	0.9476	3.0580
258–266	VQLNTSVEI	0.4873	Yes	No	0.522	17.16	0.9193	0.6880
180–189	VSFEPIPIHY	2.2860	No	No	0.4	74.16	0.9781	3.0480
99–107	VSLKCTDLK	2.5373	Yes	No	0.167	42.41	0.3644	0.5830
290–299	VTIGKIGNMR	1.1926	No	No	0.17	35.39	0.5029	1.4760
8–17	*VTVYYGVPVW*	0.0387	No	No	1.06	15.77	0.9513	1.2360
399–407	*WQKVGKAMY*	0.6072	No	No	−0.667	93.71	0.9513	2.9690
407–415	YAPPISGQI	0.6513	No	No	0.178	37.45	0.9569	0.5500
407–416	YAPPISGQIR	0.9008	Yes	No	−0.29	89.95	0.8658	1.2570

CLT epitopes were predicted using the IEDB server. Gp120 epitopes were selected based on their binding affinity to MHC-I HLAs (measured in nM), IC50 value, and antigenicity rate. The selected epitopes were further evaluated for TAP transport and Proteasomal C-terminal cleavage potential. The degree of conservation was taken into consideration for all the selected epitopes. The epitopes used in the final vaccine construct are indicated in italic form.

**Table 4:** HTL gp120 epitopes predicted using the IEDB database server.

Length	Peptide	Antigenicity	Allergenicity	Toxicity	GRAVY	IC50	IFNepitope SVM^b^
146–160	AFFYKLDIIPIDNDT	0.7514	Yes	No	0.213	6.9	Positive
288–302	AFVTIGKIGNMRQAH	1.1602	No	No	0.093	28.1	Positive
191–205	APAGFAILKCNNKTF	0.6107	No	No	0.287	99.6	Positive
177–191	CPKVSFEPIPIHYCA	1.3141	Yes	No	0.353	65	Positive
91–105	CVKLTPLCVSLKCTD	2.5249	Yes	No	0.813	23.5	Positive
125–139	*EIKNCSFNISTSIRG*	0.491	No	No	−0.24	17.3	Positive
265–269	EINCTRPNNNTRKRI	0.6071	No	No	−1.76	91	Positive
454–468	ELYKYKVVKIEPLGV	0.5524	Yes	No	0.093	13.2	Positive
183–197	EPIPIHYCAPAGFAI	0.9042	Yes	No	0.733	63.9	Positive
464–478	*EPLGVAPTKAKRRVV*	1.2335	No	No	−0.307	36.2	Positive
144–158	EYAFFYKLDIIPIDN	1.1009	No	No	0.173	7	Positive
182–196	FEPIPIHYCAPAGFA	1.1020	Yes	No	0.62	81.9	Positive
147–161	FFYKLDIIPIDNDTT	0.8371	Yes	No	0.047	11	Positive
131–145	FNISTSIRGKVQKEY	0.4398	No	No	−0.72	74.2	Positive
289–303	FVTIGKIGNMRQAHC	1.1301	No	No	0.14	78.9	Positive
148–162	*FYKLDIIPIDNDTTS*	0.8464	No	No	−0.193	25	Positive
124–138	GEIKNCSFNISTSIR	0.4983	No	No	−0.24	21.4	Positive
194–208	*GFAILKCNNKTFNGT*	0.4811	Yes	No	−0.153	84.8	Positive
139–153	*GKVQKEYAFFYKLDI*	0.7563	No	No	−0.353	18.1	Positive
413–427	GQIRCSSNITGLLLT	0.4695	No	No	0.507	96.1	Positive
286–300	GRAFVTIGKIGNMRQ	0.6843	Yes	Yes	−0.14	7.4	Positive
463–477	IEPLGVAPTKAKRRV	1.4711	No	No	−0.287	36.5	Positive
187–201	IHYCAPAGFAILKCN	0.4285	No	No	0.807	64.5	Positive
256–270	IIVQLNTSVEINCTR	0.8427	Yes	No	0.5	6.9	Positive
126–140	IKNCSFNISTSIRGK	0.6515	No	No	−0.267	11	Positive
120–134	IMEKGEIKNCSFNIS	0.5167	No	No	−0.207	93	Positive
266–280	*INCTRPNNNTRKRIR*	0.5572	No	No	−1.827	56	Positive
185–199	IPIHYCAPAGFAILK	0.5882	No	No	1.067	39.4	Positive
24–258	IRSVNFTDNAKTIIV	1.1445	No	No	0.36	32.4	Positive
411–425	ISGQIRCSSNITGLL	0.4381	Yes	No	0.547	75.5	Positive
257–271	IVQLNTSVEINCTRP	0.7967	Yes	No	0.093	11.5	Positive
18–32	KEATTTLFCASDAKA	0.61	Yes	No	−0.093	92.4	Positive
143–157	KEYAFFYKLDIIPID	1.2964	No	No	0.147	7.4	Positive
462–476	KIEPLGVAPTKAKRR	1.9736	No	No	−0.827	21.9	Positive
93–107	KLTPLCVSLKCTDLK	2.4240	Yes	No	0.36	53.6	Positive
127–141	KNCSFNISTSIRGKV	0.6432	No	No	−0.287	10.1	Positive
89–103	KPCVKLTPLCVSLKC	2.0271	No	No	0.727	11.9	Positive
254–268	KTIIVQLNTSVEINC	0.5444	No	No	0.54	5.3	Positive
401–415	KVGKAMYAPPISGQI	0.5640	Yes	No	0.087	65.8	Positive
140–154	KVQKEYAFFYKLDII	0.6655	No	No	−0.027	18.4	Positive
179–193	KVSFEPIPIHYCAPA	1.1951	Yes	No	0.307	57.6	Positive
459–473	KVVKIEPLGVAPTKA	1.5003	No	No	0.333	9.4	Positive
457–471	KYKVVKIEPLGVAPT	1.1894	Yes	No	0.127	13	Positive
466–480	LGVAPTKAKRRVVQR	0.7323	No	No	−0.5	77.1	Positive
88–102	LKPCVKLTPLCVSLK	2.0065	Yes	No	0.813	12.9	Positive
455–469	LYKYKVVKIEPLGVA	0.8394	Yes	No	0.447	5.5	Positive

The HTL epitopes were predicted using the IEDB database server. The selection of gp120 epitopes was based on their binding affinity to MHC-II HLAs (measured in nM), % rank, and antigenicity rate. All the epitopes were evaluated using the SVM method to assess their potential as IFN-γ inducers. The degree of conservation was also taken into account for all the selected epitopes. Additionally, a motif and SVM hybrid approach was used, comparing the IFN-γ inducer’s potential to those of other cytokines. The epitopes selected for inclusion in the final vaccine construct, based on their diverse immunogenic potential, are indicated in italic form.

**Table 5:** The final B-cell, MHCI, and MHCII gp120 epitopes used in the vaccine construct.

Cell Type	Peptide	Antigenicity	Allergenicity	Toxicity	GRAVY
B-Cell					
59–72	VNVTENFNMWKNDM	−0.1226	No	No	−0.821
137–148	IRGKVQKEYAFF	0.5359	No	No	−0.408
334–343	KQSSGGDPEI	0.597	No	No	−1.39
404–410	KAMYAPP	0.6789	No	No	−0.414
461–480	VKIEPLGVAPTKAKRRVVQR	1.1063	No	No	−0.39
431–458	GNSNNESEIFRPGGGDMRDNWRSELYKY	−0.0127	No	No	−1.607
CTL					
8–17	VTVYYGVPVW	0.0387	No	No	−1.06
307–315	RAKWNNTLK	0.4822	No	No	−1.7
246–254	SVNFTDNAK	1.6628	No	No	−0.789
469–478	APTKAKRRVV	0.7196	No	No	−0.71
363–372	FNSTWFNSTW	0.4342	No	No	−0.62
467–475	GVAPTKAKR	1.8579	No	No	−0.8
140–149	KVQKEYAFFY	0.6481	No	No	−0.58
142–151	QKEYAFFYKL	0.7239	No	No	−0.62
291–299	TIGKIGNMR	0.7225	No	No	−0.278
399–407	WQKVGKAMY	0.6072	No	No	−0.667
HTL					
125–139	EIKNCSFNISTSIRG	0.491	No	No	−0.24
464–478	EPLGVAPTKAKRRVV	1.2335	No	No	−0.307
148–162	FYKLDIIPIDNDTTS	0.8464	No	No	−0.193
139–153	GKVQKEYAFFYKLDI	0.7563	No	No	−0.353
266–280	INCTRPNNNTRKRIR	0.5572	No	No	−1.827
194–208	GFAILKCNNKTFNGT	0.4811	No	No	−0.153

The epitopes, including the B-cell, CTL, and HTL ones, used in the final vaccine construct were predicted using various immunoinformatic tools. The selection of these epitopes was based on several criteria, including their antigenicity, non-allergic properties, non-toxicity, and negative GRAVY values. Only the epitopes with the highest predicted immunogenic potential were included in the final vaccine construct.

**Table 6:** Different physiochemical properties of the final vaccine construct.

Features	Values
Sequence length	315 aa
Molecular weight	35,493.37 Da
Formula	C_1587_H_2443_N_465_O_444_S_11_
Antigenicity	0.6789
Theoratical pI	10.13
Total negatively charged residues	21
Total positively charged residues	52
Total number of atoms	4950
Extinction of coffiecients	60,975
Instability index	39.59
Aliphatic index	53.52
GRAVY	−0.741

## Data Availability

Data is contained within the article.
